# Human gut-microbiome-derived propionate coordinates proteasomal degradation via HECTD2 upregulation to target EHMT2 in colorectal cancer

**DOI:** 10.1038/s41396-021-01119-1

**Published:** 2022-01-01

**Authors:** Tae Young Ryu, Kwangho Kim, Tae-Su Han, Mi-Ok Lee, Jinkwon Lee, Jinhyeon Choi, Kwang Bo Jung, Eun-Jeong Jeong, Da Mi An, Cho-Rok Jung, Jung Hwa Lim, Jaeeun Jung, Kunhyang Park, Moo-Seung Lee, Mi-Young Kim, Soo Jin Oh, Keun Hur, Ryuji Hamamoto, Doo-Sang Park, Dae-Soo Kim, Mi-Young Son, Hyun-Soo Cho

**Affiliations:** 1 Korea Research Institute of Bioscience and Biotechnology, Daejeon, 34141 Republic of Korea; 2 Department of Biological Sciences, Korea Advanced Institute of Science and Technology (KAIST), Daejeon, 34141 Republic of Korea; 3 Korea University of Science and Technology, Daejeon, 34113 Republic of Korea; 4 Asan Institute for Life Sciences, Asan Medical Center and Department of Convergence Medicine, College of Medicine, University of Ulsan, Seoul, 05505 Republic of Korea; 5 Department of Biochemistry and Cell biology, School of Medicine, Kyungpook National University, Daegu, 41944 Republic of Korea; 6 Division of Molecular Modification and Cancer Biology, National Cancer Center, Tokyo, 104-0045 Japan

**Keywords:** Colorectal cancer, Transcriptomics, Functional genomics

## Abstract

The human microbiome plays an essential role in the human immune system, food digestion, and protection from harmful bacteria by colonizing the human intestine. Recently, although the human microbiome affects colorectal cancer (CRC) treatment, the mode of action between the microbiome and CRC remains unclear. This study showed that propionate suppressed CRC growth by promoting the proteasomal degradation of euchromatic histone-lysine N-methyltransferase 2 (EHMT2) through HECT domain E3 ubiquitin protein ligase 2 (HECTD2) upregulation. In addition, EHMT2 downregulation reduced the H3K9me2 level on the promoter region of tumor necrosis factor α-induced protein 1 (TNFAIP1) as a novel direct target of EHMT2. Subsequently, TNFAIP1 upregulation induced the apoptosis of CRC cells. Furthermore, using *Bacteroides thetaiotaomicron* culture medium, we confirmed EHMT2 downregulation via upregulation of HECTD2 and TNFAIP1 upregulation. Finally, we observed the synergistic effect of propionate and an EHMT2 inhibitor (BIX01294) in 3D spheroid culture models. Thus, we suggest the anticancer effects of propionate and EHMT2 as therapeutic targets for colon cancer treatment and may provide the possibility for the synergistic effects of an EHMT2 inhibitor and microbiome in CRC treatment.

## Introduction

Colorectal cancer (CRC) is a commonly diagnosed cancer worldwide. Although chemotherapy (5-fluorouracil (5-FU) and oxaliplatin) and targeted therapy (cetuximab and bevacizumab) are used to treat this disease, new therapeutic methods are needed to reduce the side effects and increase the success rate of CRC treatment [1–3]. Recently, the human microbiome, including metabolites and the microbiome, has uncovered alternative new CRC treatments [4–6].

In the human body, the microbiome mainly colonizes the large intestine, promotes food digestion, regulates the human immune system, produces vitamins (B12, K, riboflavin), and protects against disease-related bacteria [7–9]. Short-chain fatty acids (SCFAs; e.g., butyrate, acetate, and propionate) are metabolic products produced via dietary fiber fermentation by the microbiome in the colon [10] and are involved in insulin resistance and colonic inflammation [11]. Recently, in colon cancer, we reported that propionate suppresses CRC growth by downregulating protein arginine N-methyltransferase 1 (PRMT1) expression, and PRMT1 reduction induced cell apoptosis by controlling the mTOR pathway [12]. However, the mode of action (MOA) of propionate for CRC suppression remains incompletely understood, particularly regarding epigenetics.

In colon cancer, the regulation of histone methylation and demethylation is an essential process to regulate tumor suppressor and oncogene expression [13–15]. Several histone methyltransferases and demethylases are overexpressed and involved in CRC growth and metastasis via epigenetic regulation [16, 17]. Among them, euchromatic histone-lysine N-methyltransferase 2 (EHMT2) performs mono- and dimethylation of histone H3 lysine 9 to generate heterochromatin for the repression of tumor suppressor genes. Knockdown of EHMT2 by small interfering RNA (siRNA) resulted in cell apoptosis, cell cycle arrest, and metastasis in several types of cancer [18–20]. Although EHMT2 is an important therapeutic target for CRC treatment, the regulation of EHMT2 expression in CRC, particularly the relationship between EHMT2 and the human microbiome, remains elusive.

Thus, in this study, we demonstrated the anticancer effect of propionate at the epigenetic level. Propionate treatment upregulated HECT domain E3 ubiquitin protein ligase 2 (HECTD2) expression to promote the proteasomal degradation of EHMT2 via posttranslational modifications. Subsequently, EHMT2 downregulation by propionate-induced tumor necrosis factor α-induced protein 1 (TNFAIP1) expression by reducing H3K9me2 levels to promote colon cancer apoptosis. In addition, we confirmed this mechanism using *Bacteroides thetaiotaomicron* (BT) and *Collinesella aerofaciens* (CA) culture medium. Finally, using 3D spheroid CRC models, we identified EHMT2 as a therapeutic target for colon cancer treatment with propionate.

## Materials and methods

### Cell culture and reagents

The human CRC cell lines HCT116 and LS174T were purchased from the Korean Cell Line Bank and cultured in RPMI-1640 medium supplemented with 10% fetal bovine serum (FBS) and 1% penicillin/streptomycin in a humidified atmosphere with 5% CO_2_ at 37 °C. Sodium propionate (SP; P5436), sodium butyrate (SB; 303410), sodium acetate (SA; 71183), and sodium D-lactate (SL; 71716) were purchased from Sigma-Aldrich. MG132 (M7449) and cycloheximide (CHX; C4859) were purchased from Sigma-Aldrich. BIX01294 (ab141407) was purchased from Abcam.

### Bacterial culture

*Bacteroides thetaiotaomicron* DS1880 and *Collinesella aerofaciens* KGM02679 were obtained from the Bio R&D Product program (https://biorp.kribb.re.kr/) and Koran Gut Microbiome Bank (https://www.kobic.re.kr/kgmb_dist/), respectively. The bacterial strains were cultivated in tryptic soy broth (BD, Sparks, MD, USA) with 5% horse blood under anaerobic conditions at 37 °C for 36 h. To achieve anaerobic conditions, the liquid medium was purged with ultrapure nitrogen gas before autoclaving for 15 min, and the residual oxygen was removed by keeping the medium air permeable in an anaerobic chamber where the oxygen concentration was controlled to 0 ppm when measured with a CAM-12 anaerobic monitor (Coy Laboratory Products, Grass Lake, MI, USA) after adding horse blood to the sterilized medium. The bacterial cultures were incubated at 65 °C for 30 min and centrifuged at 3000 g for 10 min. The supernatants were collected in a new tube and kept at –70 °C until use.

### Cell viability assay

Cell Counting Kit-8 (CCK-8; Dojindo Laboratories) was used to conduct the cell viability assays. Cells were seeded in 6-well plates at 4 × 10^5^ cells/well and incubated for 24 h. After SP, SB, SA, SL, BT sup, and CA sup treatment or siRNA transfection for 48 h or BIX01294 treatment for 24 h, CCK-8 solution and RPMI-1640 medium with 10% FBS were added to each well and incubated with 5% CO_2_ at 37 °C for 2 or 5 min. The absorbance was assessed using a microplate reader at 450 nm. For crystal violet (CV) staining, the cells were fixed with methanol for 5 min and stained with 0.1% CV after SP, SB, SA, SL, BT sup, and CA sup treatment or siRNA transfection for 48 h or BIX treatment for 24 h.

### Fluorescence-activated cell sorting (FACS) analysis

After treatment with SP, SB, SA, SL, BT sup, and CA sup or siRNA transfection for 48 h or BIX for 24 h, the cells were collected and incubated with the Muse Annexin V and Dead Cell Assay kit (MCH100105; Merck) for 20 min at room temperature. For analysis using the Muse™ Caspase 3/7 Kit (MCH100108; Merck), the cells were incubated with caspase 3/7 reagent (Merck) for 30 min in a humidified atmosphere with 5% CO_2_ at 37 °C. After incubation, the cells were incubated with Caspase 7-AAD (Merck) for 5 min at room temperature. After incubation, ~5 × 10^4^ cells were analyzed using a Muse Cell analyzer (Merck). The FACS results were analyzed using Muse 1.6 Analysis software (Merck).

### siRNA transfection

For siRNA transfection, HCT116 and LS174T cells were seeded in plates and incubated for 24 h. The targeting or control siRNAs (Bioneer Co., Ltd) were transfected into cancer cell lines at 100 nM using RNAiMax (Invitrogen) for 48 h [21]. The sequences of siRNAs are listed in Supplementary Table S1.

### Semiquantitative reverse transcription PCR and quantitative real-time PCR

Total RNA was isolated from the indicated cell lines using a Qiagen RNeasy Mini kit (Qiagen) according to the manufacturer’s instructions. RNA aliquots of 1 μg were then reverse-transcribed using the iScript cDNA synthesis kit (Bio-Rad) according to standard protocols provided by the manufacturer. For semiquantitative RT-PCR, cDNA was used as a template for PCR using AccuPower HotStart PCR PreMix (Bioneer). Quantitative RT-PCR (EHMT2: annealing temperature 55 °C, 35 cycles; ACTB: annealing temperature 58 °C, 28 cycles) was performed using the SimpliAmp Thermal Cycler (Applied Biosystems) following the manufacturer’s instructions. Quantitative real-time PCR was performed on cDNA samples using Brilliant III Ultra-Fast SYBR Green QPCR Master Mix, and the signal was detected using the AriaMx Real-Time PCR System (both from Agilent Technologies). The fluorescence threshold value was calculated using Agilent Aria 1.6 software (Agilent Technologies) [20]. The primers used for semiquantitative RT-PCR and quantitative real-time PCR are listed in Supplementary Table S2.

### Western blot analysis

The cells were washed once with phosphate-buffered saline (PBS) and then lysed in cold lysis buffer (50 mM Tris-HCl at pH 7.4, 150 mM NaCl, 1% Triton X-100, 0.1% SDS, 1 mM EDTA, 1 mM Na3VO4, 1 mM NaF, and 1× protease inhibitor cocktail). Cell lysates were centrifuged at 14,000 × *g* for 20 min at 4 °C and then boiled in 5× sample buffer following protein determination (BSA, 23208; Thermo Fisher Scientific). Histone was purified from the indicated cells using a Histone Extraction Kit (40028; Active Motif) according to the manufacturer’s instructions. The protein samples were subjected to western blot analysis, in which nitrocellulose membranes (1620145; Bio-Rad), blocking reagent (5% skim milk, 1 h at room temperature), and 4–20% precast gels (456–1094; Bio-Rad) were used with the indicated antibodies at a 1:1000 dilution ratio [8]. The membranes were stained with the following primary antibodies: EHMT2 (ab185050), TNFAIP1 (ab86934), HECTD2 (ab173572), H3K9ac (ab4441), and H3K27ac (ab4729) from Abcam, H3 (4620), and PARP (9542) from Cell Signaling Technology, FLAG (F1804) from Sigma-Aldrich, and HA (sc-805) and ACTB (sc-47778) from Santa Cruz Biotechnology. The membranes were then incubated with secondary antibodies (rabbit; sc-2357, mouse; sc-2031; Santa Cruz Biotechnology) at room temperature for 1 h, and ECL solution (170–5060; Bio-Rad) was used for visualization. A chemiluminescence imaging system (Mini HD9; UVitec) was used for imaging. Quantification of the western blots was performed using ImageJ. The relative quantification values were calculated as the ratio of the protein bands and loading control.

### Immunoprecipitation

pCAGGS-n3FC (Mock), pCAGGS-n3FCEHMT2 (3× FLAG-EHMT2), and Ub-HA were transfected into HCT116 cell lines using FuGENE 6 Transfection Reagent (Promega) for 48 h. Transfected HCT116 cells were lysed with cold lysis buffer (50 mM Tris-HCl at pH 7.4, 150 mM NaCl, 1% Triton X-100, 0.1% SDS, 1 mM EDTA, 1 mM Na3VO4, 1 mM NaF, and 1× protease inhibitor cocktail). In a typical immunoprecipitation reaction, 800 μg of whole-cell extract was incubated with ANTI-FLAG M2 Affinity Gel (A2220; Sigma-Aldrich). After the beads were washed three times in 1 ml of Tris-buffered saline (pH 7.6), proteins that bound to the beads were eluted by boiling in 2× sample buffer (Thermo Scientific).

### Chromatin immunoprecipitation assay

Chromatin immunoprecipitation (ChIP) assays were performed using Magna ChIP A/G (Magna0013 and Magna0014; Millipore) following the manufacturer’s instructions. HCT116 cells were transfected with siCont and siEHMT2 for 48 h or treated with DMSO and BIX01294 for 24 h, crosslinked with 1% formaldehyde (Sigma-Aldrich) for 10 min at room temperature, and quenched with 1× glycine (Millipore) for 5 min at room temperature. Next, HCT116 cells were washed with cold 1× PBS (containing 1× Protease inhibitor Cocktail II). After nuclear extraction, the chromatin solution was sonicated using a Bioruptor Pico sonication device (B01060010; Diagenode) with 15 cycles of 30 s on and 30 s off to obtain 200- to 1000-bp chromatin fragments. Sheared chromatin was incubated with 2 μg of H3K27ac (ab4729; Abcam), 2 μg of H3K9me2 (ab1220; Abcam) antibody, or 2 μg of H3K9ac (ab4441; Abcam) antibody with 20 μl of Magna ChIP A/G magnetic beads (Millipore) overnight at 4 °C. The complexes were incubated with ChIP elution buffer and RNase A mixture for 30 min at 37 °C and then incubated with proteinase K for 2 h at 62 °C. After DNA purification using spin columns, the samples were analyzed by quantitative real-time PCR. The primers used for the ChIP assay are listed in Supplementary Table S3.

### Immunocytochemistry

Cultured cells were fixed in 4% paraformaldehyde at room temperature for 10 min, permeabilized in 0.5% Triton X-100 (Sigma-Aldrich) in PBS for 10 min, and blocked with 5% bovine serum albumin in PBS for 30 min. Fixed cells were incubated with anti-EHMT2 antibody (ab185050; Abcam) and anti-TNFAIP1 antibody (ab86934; Abcam) overnight at 4 °C and stained with Alexa Fluor-conjugated secondary antibodies (Life Technologies). Fluorescent images were obtained using a CELENA S Digital Imaging System (Logos Biosystems).

### Immunohistochemistry

Paraffin-embedded sections of a colon tumor tissue array (T8235722–2, Biochain) were processed in a microwave (90 °C) with antigen-retrieval solution (pH 9) (S2367; Dako), treated with a peroxidase-blocking reagent, and then treated with a protein-blocking reagent (K130, X0909; Dako). The tissue sections were incubated with rabbit anti-EHMT2 antibody (68851; CST) followed by incubation with an HRP-conjugated secondary antibody (Dako). Immunoreactivity was visualized using a chromogenic substrate (Liquid DAB Chromogen; Dako). Finally, the tissue specimens were stained with Mayer’s hematoxylin solution (Hematoxylin QS; Vector Laboratories) for 5 s to discriminate the nucleus from the cytoplasm [22].

### RNA-seq and analysis

Using the TruSeq RNA Sample Preparation Kit V2, purification and library construction were performed using total RNA, and HiSeq 2500 machines (Illumina, San Diego, CA, USA) were used for sequencing using a read length of 2 × 100 bases. FastQC v.0.11.4 was used to determine the quality of the paired-end reads. Cutadapt v.1.15 and Sickle v. 1.33 were used to filter low-quality reads and adaptors. Cufflinks version 2.2.1 was used to calculate FPKM (fragments per kilobase of transcripts per million mapped reads) values. Cuffdiff was used to select differentially expressed genes (DEGs) (fold change > 2). All Gene Ontology (GO) and KEGG pathway enrichment analyses were performed using Database for Annotation, Visualization and Integrated Discovery (DAVID) ver. 6.8 and CluGO ver. 2.5.5 in Cytoscape ver. 3.7.1. Gene set enrichment analysis (GSEA) was performed using GSEA ver. 4.0.1.

### Mouse experiments

To establish a xenograft mouse model, HCT116 cells were implanted into the flanks of 6-week-old Balb/c-nu female mice (Orient Bio., Sungnama, Korea). BIX01294 was intraperitoneally injected into mice three times a week. Tumor size was measured twice every week, and the tumor volume was calculated as length (L) × width (W) × height (H). On day 23, all the mice were sacrificed. The animal experiments were approved by the Committee on Animal Experimentation of the Korea Research Institute of Bioscience and Biotechnology.

### 3D spheroid culture

To perform spheroid culture of HCT116 cell lines, ultralow attachment (ULA) microplates were used (Corning; Cat. 7007). HCT116 cells were seeded into plates at concentrations of 5 × 10^4^ cells per well and incubated for 24 h. Subsequently, SP was treated and cultured for 4 days. To evaluate the synergistic effect, SP and BIX01294 were treated and cultured for 2 days, and the spheroids were observed under a microscope (CKX53; Olympus Corporation).

### Differentiation of human intestine organoids (hIOs)

hIOs were generated according to a previously reported protocol. To induce definitive endoderm (DE) identity from human embryonic stem cells (hESCs), hESCs were treated with 100 ng/ml Activin A (R&D Systems, Minneapolis, MN, USA) and increasing concentrations of 0, 0.2, and 2% FBS for 3 days. Next, the DE cells were treated with 500 ng/ml FGF4 (Peprotech, Rocky Hill, NJ, USA), 3 μM CHIR99021 (Tocris, Ellisville, MO, USA), and 2% FBS for differentiation into the hindgut (HG). The spontaneously generated 3D HG spheroids were embedded in Matrigel (BD Biosciences, San Diego, CA, USA) domes and cultured in hIO culture medium containing 1× B27 supplement (Thermo Fisher Scientific Inc., Waltham, MA, USA), 100 ng/ml EGF (R&D Systems), 500 ng/ml R-spondin1 (Peprotech), and 100 ng/ml Noggin (R&D Systems). The hIO culture medium was replaced every 2 days, and the HIOs were passaged every 10 days. The hIOs were treated with 1 mM, 2.5 mM, and 5 mM propionate (Sigma-Aldrich, St. Louis, MO, USA) in hIO medium for two passages.

### Cell viability assay of hIO

Cell viability was determined using the EZ-CYTOX Cell Viability, Proliferation & Cytotoxicity assay Kit (DOGENBIO, Seoul, Korea) according to the manufacturer’s instructions. Control and propionate-treated hIOs were incubated with 200 µl of hIO culture medium containing 10% EZ-CYTOX reagent in a 37 °C incubator for 3 h. After incubation, 50 µl of supernatant was transferred to 96-well plates, and the absorbance was measured at 450 nm using a Spectra Max M3 microplate reader (Molecular Devices, Sunnyvale, CA, USA). The results were normalized to the total cell number of hIOs.

### Statistical analysis

The results are expressed as the means ± SDs (error bars) of three independent experiments. Student’s *t*-test and two-way ANOVA were performed using GraphPad Prism 5.0 (GraphPad Software, Inc., La Jolla, CA, USA). The cutoff for significance was *p* < 0.05.

## Results

### Propionate treatment induces apoptosis in HCT116 and LS174T cells

**Fig. 1:**
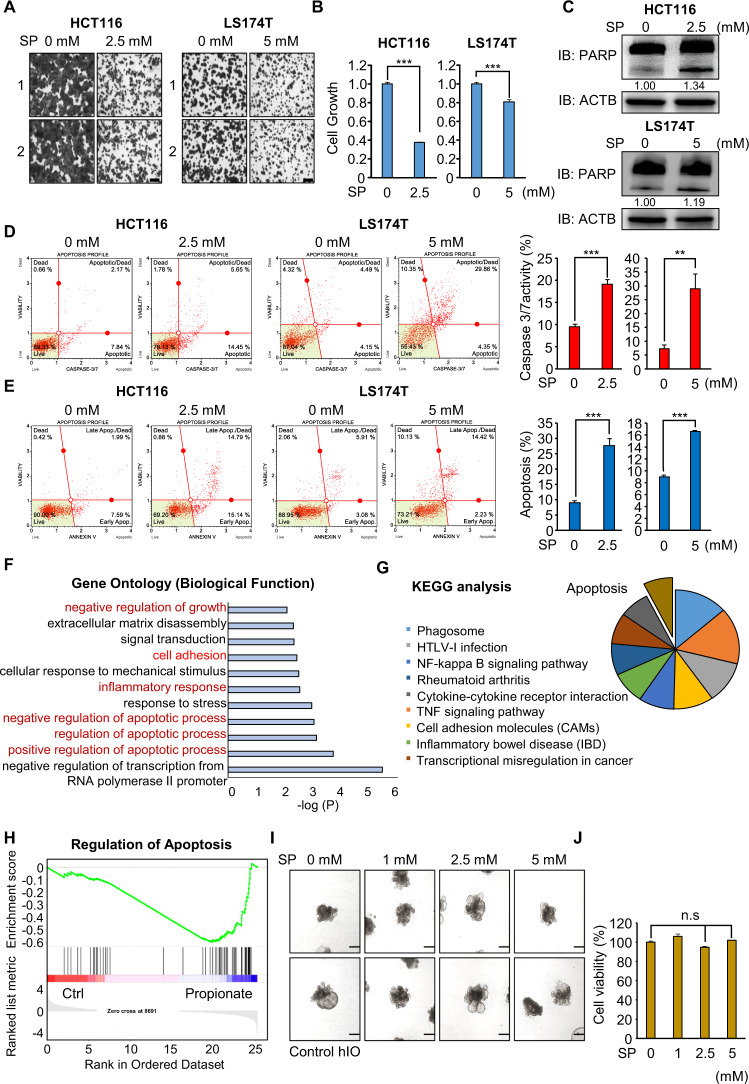
Propionate suppresses the cell growth of HCT116 and LS174T cells via cell apoptosis. **A**, **B** Cell growth assay after sodium propionate treatment for 48 h. HCT116 and LS174T cells were fixed in 100% methanol and stained with crystal violet solution. Scale bar, 200 μm (**A**). Cells were incubated for 5 min at 37 °C after adding CCK-8 solution. The intensity of cell growth was measured using a microplate reader (450 nm). Mean ± SD of three independent experiments. *p* values were calculated using Student’s *t*-test (***p < 0.001) (**B**). **C** Western blot analysis after sodium propionate treatment using anti-PARP. ACTB was used as the internal control in HCT116 and LS174T cells. The signal intensities were quantified using ImageJ software. **D** FACS analysis using Muse Caspase 3/7 working solution was performed after sodium propionate treatment. The upper right panel indicates the apoptotic and dead cell proportions (left). Quantification of caspase 3/7 activity. Mean ± SD of three independent experiments. *p* values were calculated using Student’s *t*-test (***p < 0.001, **p < 0.01) (right). **E** FACS analysis of Annexin V staining was performed after sodium propionate treatment. The lower right and upper right quadrants indicate early apoptosis and late apoptosis (left). Quantification of apoptosis. Mean ± SD of three independent experiments. *p* values were calculated using Student’s *t*-test (***p < 0.001) (right). **F** DAVID-based GO analysis of RNA-seq results of the up- and downregulated genes among the 872 genes. **G** DAVID-based KEGG pathway analysis of 872 genes. The enriched terms are shown. **H** GSEA using 872 genes. **I** Representative images of the morphology of hIOs after treatment with varying concentrations of propionate. Scale bar, 500 μm. **J** Cell viability assessed by the CCK assay in hIO treated with the indicated concentrations of propionate. *n* = 3 per group. Mean ± SD of three independent experiments. *p* values were calculated using Student’s *t*-test (n.s. nonsignificant).

To assess the anticancer effects of propionate in colon cancer, we first calculated the IC_50_ concentration of SP in HCT116 and LS174T cell lines, revealing 2.986 mM (HCT116) and 11.67 mM (LS174T) reaction concentrations (Supplementary Fig. S1). Thus, we selected 2.5 mM (HCT16) and 5 mM (LS174T) SP to evaluate the MOA of propionate-induced apoptosis in colon cancer. Next, we performed a cell growth assay using HCT116 and LS174T cells after SP treatment. As shown by CV staining and CCK-8 assays, cell growth was significantly suppressed in the SP treatment groups compared with that in the negative control groups (Fig. 1A, B). In addition, western blot analysis demonstrated that the levels of cleaved PARP were increased in HCT116 and LS174T cells treated with SP (Fig. 1C). Furthermore, FACS analysis revealed the induction of caspase 3/7 activity in CRC cell lines after SP treatment (Fig. 1D) as well as the induction of early and late apoptosis (Fig. 1E). In RNA-seq analysis of HCT116 cells treated with SP, 398 upregulated genes and 474 downregulated genes were detected (over two-fold). Using DEGs, we performed GO analysis (biological processes) using the CluGO plug in Cytoscape (ver 3.7.1.) and found that propionate treatment was related to the cell growth GO terms “negative regulation of biological process” and “positive regulation of cell death” (Supplementary Fig. S2). In addition, using the DAVID, we found enrichment of the negative regulation of growth, positive regulation of apoptosis, cell adhesion, and inflammatory response (Fig. 1F). Moreover, KEGG pathway analysis revealed that propionate treatment affected apoptosis, the TNF signaling pathway, and transcriptional misregulation in cancer (Fig. 1G). GSEA found that apoptosis was regulated by SP treatment compared with the control group (Fig. 1H).

To assess the toxicity of propionate in the normal intestine, we generated human pluripotent stem cell (hPSC)-derived intestinal organoids (hIOs) and performed a viability test in a dose-dependent manner with propionate (0 mM, 1 mM, 2.5 mM, 5 mM). hPSC-derived hIO more closely mimics the culture environment and structural complexity of the human intestine, and hIO will likely be a successful future model for disease modeling and drug screening in pharmacological and toxicological industries [22–26]. Propionate treatment did not affect the viability of human intestinal organoids treated with several concentrations of propionate (Fig. 1I, J and Supplementary Fig. S3). Thus, through hIO experiments, we suggest that the reaction concentration of propionate in this study effectively suppressed the viability of colon cancer cell lines but not the normal human intestine.

### EHMT2 is a target for propionate-induced cell apoptosis in colon cancer

**Fig. 2:**
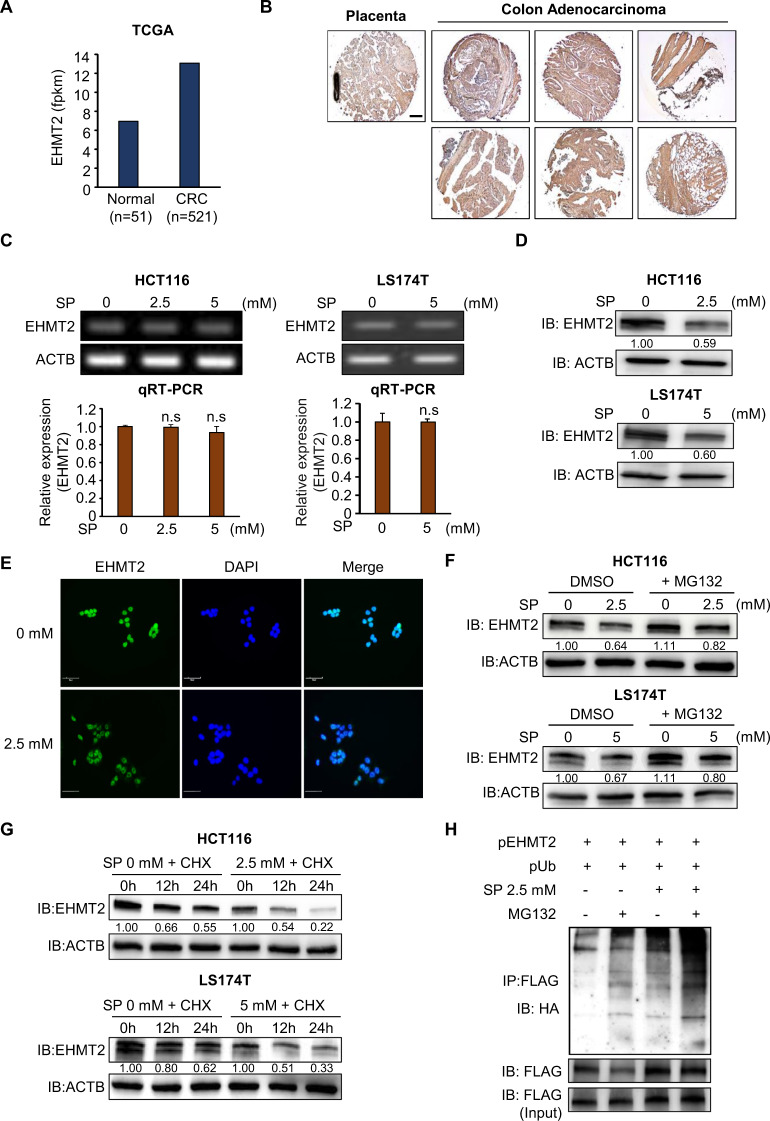
EHMT2 is a target for propionate-induced cell apoptosis in colon cancer. **A** The expression of EHMT2 in normal and CRC samples derived from the TCGA portal. **B** Immunohistochemical analysis of EHMT2. A colon cancer tissue microarray was purchased from BioChain (https://www.biochain.com). Scale bar, 200 μm. **C** Expression level of EHMT2 after sodium propionate treatment in HCT116 and LS174T cells. RT-PCR analysis of EHMT2. Actin (ACTB) was used as an internal control (upper). qRT-PCR analysis of EHMT2 expression. Mean ± SD of three independent experiments. *p* values were calculated using Student’s *t*-test (n.s. nonsignificant) (below). **D** Western blot analysis after sodium propionate treatment using anti-EHMT2. ACTB was used as the internal control in HCT116 and LS174T cells. The signal intensities were quantified using ImageJ software. **E** Immunocytochemical analysis of EHMT2. HCT116 cells treated with sodium propionate were fixed with 100% methanol and stained with anti-EHMT2 (Alexa Fluor 488, green) and DAPI (blue). Scale bar, 50 μm. **F** Western blot analysis after SP treatment for 24 h and MG132 for 6 h using anti-EHMT2. ACTB was used as the internal control in HCT116 and LS174T cells. The signal intensities were quantified using ImageJ software. **G** Western blot analysis of EHMT2 after SP treatment for 24 h and cycloheximide (CHX) for 12 and 24 h. ACTB was used as the internal control. The signal intensities were quantified using ImageJ software. **H** SP induced ubiquitination of EHMT2. After transfection with a FLAG-tagged EHMT2 expression vector together with HA-tagged ubiquitin and SP treatment for 24 h and MG132 for 6 h, western blot analysis was performed using HA and FLAG antibodies.

Next, to identify the effect of cell apoptosis induced by SP treatment, we focused on histone-lysine methyltransferases (HMTs) and demethylases (HDLs) because several types of HMTs and HDLs are overexpressed in CRC, and the knockdown of HMTs and HDLs suppresses CRC proliferation via CRC cell apoptosis [16, 17]. Using in silico epigenetic panels, we selected the overexpression of HMTs and HDLs (1.5-fold, colon cancer/normal) in 521 colon cancer and 51 normal samples derived from the TCGA data portal (https://tcga-data.nci.nih.gov/). We selected EHMT2 as the effector gene of SP-induced apoptosis in CRC (Fig. 2A and Supplementary Fig. S4). In histochemical analysis using a CRC tissue microarray, the expression level of EHMT2 was induced in CRC tissues compared with normal placental tissues (Fig. 2B). EHMT2 is an HMT involved in H3K9 dimethylation to form heterochromatin in transcriptional gene regulation. In addition, in breast, bladder, and lung cancer, EHMT2 knockdown was involved in cell apoptosis and cell migration/invasion [18–20, 27]. After treatment of the CRC cell lines with SP, we detected no changes in EHMT2 at the transcriptional level; however, in western blot analysis, EHMT2 expression was significantly reduced (Fig. 2C, D). In addition, in immunocytochemical (ICC) analysis after SP treatment, the intensity of EHMT2 in the SP-treated cells was significantly decreased compared with that in the control cells (Fig. 2E). Therefore, we hypothesized that propionate might be involved in translational modification and proteasomal degradation. Next, to verify whether SP treatment induced the proteasomal degradation of EHMT2, we performed western blot analysis after cotreatment with SP and MG132, an inhibitor of proteasomal degradation, in HCT116 and LS174T cells. In the cotreatment group, reduced EHMT2 expression by SP treatment was recovered by MG132 treatment compared with the control, suggesting that SP induced the proteasomal degradation of EHMT2 (Fig. 2F). In addition, after treatment with CHX, a protein synthesis inhibitor, we detected an increase in the protein degradation induced by SP treatment compared with that in the control group in the CRC cell lines (Fig. 2G). Finally, to assess the status of EHMT2 polyubiquitination by SP, we cotransfected FLAG-tagged EHMT2 and HA-tagged ubiquitin into HCT116 cells treated with SP and/or MG132 and performed immunoprecipitation experiments using an anti-FLAG antibody. EHMT2 polyubiquitination in the SP and MG132 treatment groups was significantly induced compared with that in the control groups (Fig. 2H). Thus, propionate affected EHMT2 stability by regulating posttranslational modification for proteasomal degradation; consequently, the CRC cell lines suppressed cell proliferation and induced cell apoptosis. This report is the first to describe a relationship between propionate treatment and translational modification.

### SP treatment induces HECTD2 expression to degrade EHMT2

**Fig. 3:**
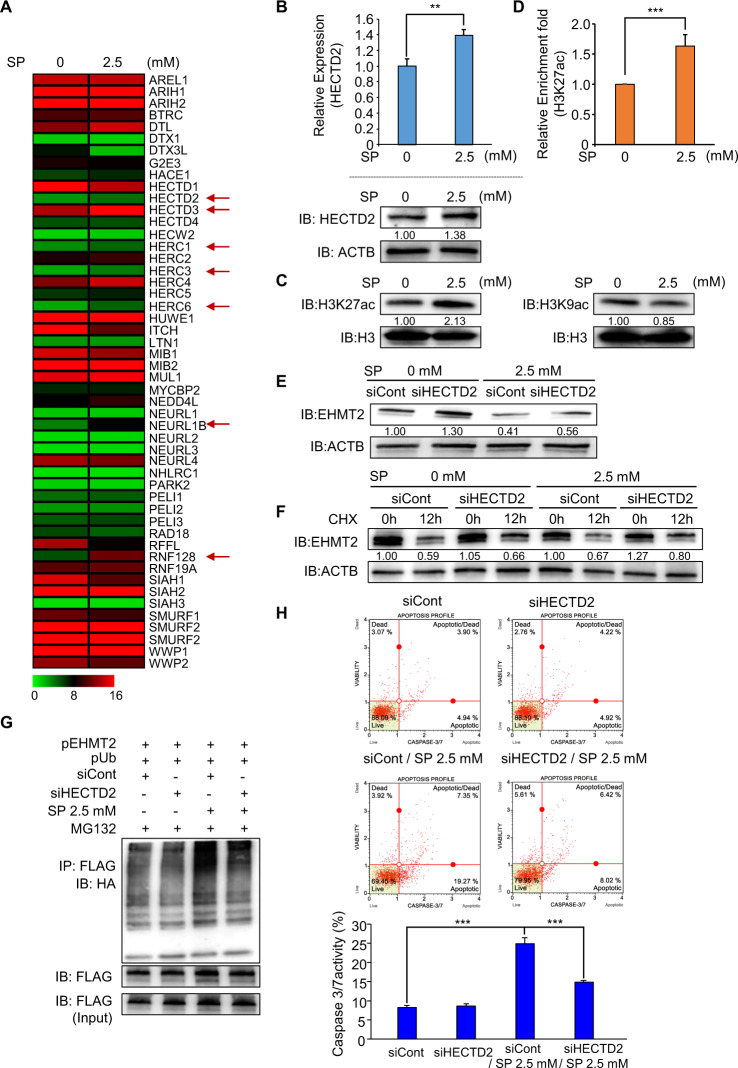
SP treatment induces HECTD2 expression for EHMT2 degradation. **A** Heat map of RNA-seq data (E3 ubiquitin ligase) in the control and SP treatments. **B** qRT-PCR analysis of HECTD2 after SP treatment. Mean ± SD of three independent experiments. *p* values were calculated using Student’s *t*-test (**p < 0.01, ***p < 0.001) (upper). Western blot analysis after sodium propionate treatment using anti-HECTD2. ACTB was used as the internal control in HCT116 cells (below). The signal intensities were quantified using ImageJ software. **C** Western blot analysis of histone H3K27 acetylation (left) and H3K9 acetylation (right) after SP treatment for 24 h in HCT116 cells. Histone H3 was used as the internal control. The signal intensities were quantified using ImageJ software. **D** The ChIP assay was performed using anti-H3K27ac antibody. The result is shown as an enrichment fold of input chromatin compared with the control in HCT116 cells after SP treatment. Mean ± SD of three independent experiments. *p* values were calculated using Student’s *t*-test (***p < 0.001). **E** Western blot analysis of EHMT2 after treatment with siHECTD2 and SP for 24 h in HCT116 cells. ACTB was used as the internal control. The signal intensities were quantified using ImageJ software. **F** Western blot analysis of EHMT2 after treatment with siHECTD2 and SP for 24 h and CHX for 12 h in HCT116 cells. ACTB was used as the internal control. The signal intensities were quantified using ImageJ software. **G** Western blot analysis after transfection with a FLAG-tagged EHMT2 expression vector together with HA-tagged ubiquitin and treatment with siHECTD2 and SP for 24 h and MG132 for 6 h using HA and FALG antibodies in HCT116 cells. **H** FACS analysis using Muse Caspase 3/7 working solution was performed after cotreatment with HECTD2 knockdown and SP in HCT116 cells. The upper right panel indicates the apoptotic and dead cell proportions (upper). Quantification of caspase 3/7 activity. Mean ± SD of three independent experiments. *p* values were calculated using Student’s *t*-test (***p < 0.001) (below).

To assess the mechanism for EHMT2 degradation by SP treatment, we reanalyzed the RNA-seq data (49 E3 ubiquitin ligases selected) and found upregulation of 7 E3 ligases (HECTD2, HECTD3, HERC1, HERC6, NEURL1B, and RNF128) by SP treatment compared with the control treatment (Fig. 3A). To verify the relationship between proteasomal degradation and E3 ligase, we performed recovery analysis after treatment with siRNAs and SP and finally observed that HECTD2 knockdown helped rescue the degradation of EHMT2 induced by SP treatment (Supplementary Fig. S5). In qRT-PCR and western blot analysis, we reconfirmed the upregulation of HECTD2 expression by SP treatment (Fig. 3B). SCFAs, such as butyrate and propionate, inhibited HDAC activity in cancer cells [28, 29]; thus, we performed western blot analysis after treatment with SP in HCT116 cells. The H3K27 acetylation status was increased by SP treatment compared with that of the control; however, the H3K9 acetylation status was not changed by SP treatment (Fig. 3C). Therefore, we selected H3K27 acetylation to evaluate HECTD2 expression by SP treatment. Next, we performed a ChIP assay using a histone H3K27 acetylation antibody after SP treatment in HCT116 cells, revealing the induction of the H3K27 acetylation status in promoter regions of HECTD2 (Fig. 3D). Our findings suggest that microbiome-induced propionate inhibits the activity of HDAC to upregulate HECTD2 expression in colon cancer.

HECTD2 is an E3 ligase that is recognized as a substrate for ubiquitin transfer from the E2 ubiquitin-conjugating enzyme [30]. In siRNA analysis of HECTD2, the degradation of EHMT2 by SP was inhibited by HECTD2 knockdown (Fig. 3E). In CHX treatment analysis, the degradation rate of EHMT2 by SP was reduced by siHECTD2 treatment compared with the control siRNA treatment (Fig. 3F). In addition, we detected a reduction in the polyubiquitination status in the siHECTD2 and SP treatment groups (Fig. 3G). Furthermore, FACS analysis of caspase 3/7 activity showed that cotreatment with siHECTD2 and SP decreased caspase 3/7 activity compared with SP treatment only (Fig. 3H). Thus, the induction of HECTD2 expression by propionate may promote the proteasomal degradation of EHMT2; accordingly, the growth of colon cancer was suppressed by apoptosis induced by EHMT2 reduction.

### Knockdown of EHMT2 is involved in the apoptosis of CRC cells

**Fig. 4:**
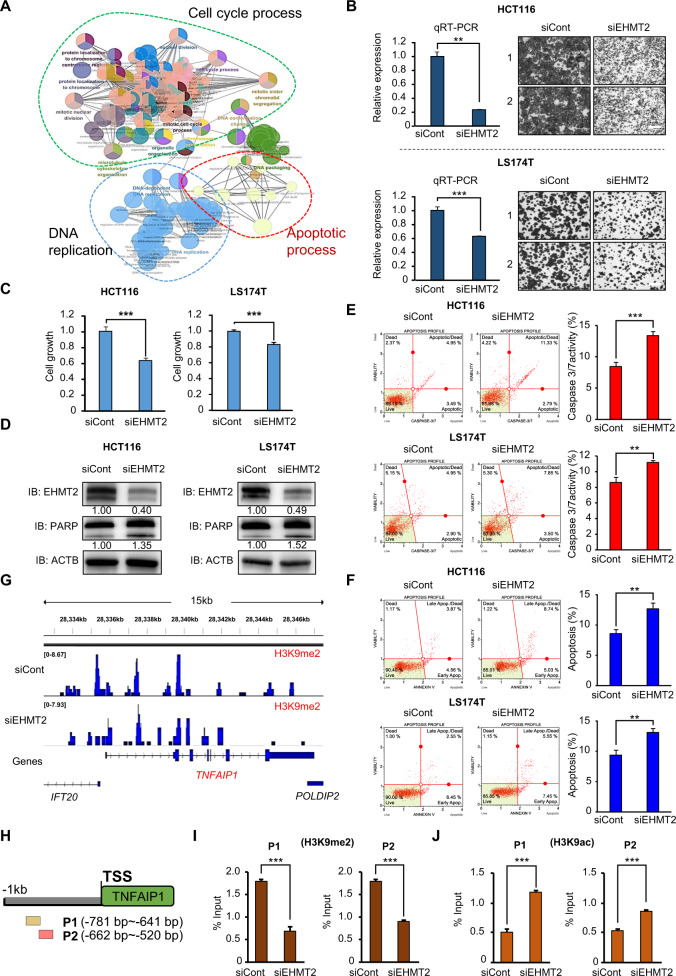
Knockdown of EHMT2 is involved in CRC apoptosis. **A** GO pathway term enrichment networks. GO pathway term networks in the EHMT2 knockdown and control groups were functionally grouped by ClueGO. Terms in the functionally grouped networks were cut off at *p* values > 0.05. **B** qRT-PCR analysis of EHMT2 after treatment with EHMT2 siRNA and siCont (negative control). *p* values were calculated using Student’s *t*-test (**p < 0.01) (left). Cell growth assay after treatment with siEHMT2 and siCont for 48 h. HCT116 and LS174T cells were fixed in 100% methanol and stained with crystal violet solution. Scale bar, 200 μm (right). **C** CCK-8 assay. CCK-8 solution was added to the culture medium after treatment with siEHMT2 and siCont, and the cells were incubated for 5 min at 37 °C. The intensity of cell growth was measured using a microplate reader (450 nm). *p* values were calculated using Student’s *t*-test (***p < 0.001). **D** Western blot analysis after EHMT2 knockdown using anti-EHMT2 and anti-PARP antibodies. ACTB was used as the internal control in HCT116 and LS174T cells. The signal intensities were quantified using ImageJ software. **E** FACS analysis using Muse Caspase 3/7 working solution was performed after EHMT2 knockdown. The upper right panel indicates the apoptotic and dead cell proportions (left). Quantification of caspase 3/7 activity. Mean ± SD of three independent experiments. *p* values were calculated using Student’s *t*-test (***p < 0.001, **p < 0.01) (right). **F** FACS analysis of Annexin V staining was performed after EHMT2 knockdown. The lower right and upper right quadrants indicate early apoptosis and late apoptosis (left), respectively. Quantification of apoptosis. Mean ± SD of three independent experiments. *p* values were calculated using Student’s *t*-test (**p < 0.01) (right). **G** ChIP-seq analysis. Representative IGV view of the H3K9me2 (siCont) and H3K9me2 (siEHMT2) ChIP signals in the promoter region of a TNFAIP1 gene. The promoter region was defined as the region from the transcription start site (TSS) to 1 kb upstream of the TSS. The Y-axis scale reflects the normalized number of reads using RPGC (number of reads per 10 bp/scaling factor for 1× average coverage) of deepTools (v3.3.0). The normalized scales are 0–8.67 for H3K9me2 (siCont) and 0–7.93 for H3K9me2 (siEHMT2). **H** Graphical abstract for ChIP primer design on the TNFAIP1 promoter region. **I**, **J** The ChIP assay was performed using anti-H3K9me2 (**I**) and anti-H3K9ac (**J**) antibodies. The results are expressed as a percentage of input chromatin compared with the control in HCT116 cells after siEHMT2 treatment. Mean ± SD of three independent experiments. *p* values were calculated using Student’s *t*-tests (***p < 0.001).

The RNA-seq results after EHMT2 knockdown showed that the apoptotic process, cell cycle, and regulation of the mitotic cell cycle were enriched in GO analysis compared with those after siCont treatment (Fig. 4A). To assess whether EHMT2 downregulation by SP treatment induced cell apoptosis, we designed EHMT2-specific siRNA and performed a cell growth assay after treatment with the EHMT2 siRNA. Cell growth in the CRC cell lines determined by CV staining and CCK-8 assays was suppressed by EHMT2 knockdown compared with that in the siCont group, as demonstrated by SP treatment (Fig. 4B, C). Western blot analysis showed that cleaved PARP was increased by EHMT2 knockdown (Fig. 4D). In FACS analysis for caspase 3/7 activity and apoptosis, the EHMT2 knockdown group showed upregulation of caspase 3/7 activity in the CRC cell lines (Fig. 4E). The population of early and late apoptotic cells was increased by EHMT2 knockdown compared with that in the siCont group (Fig. 4F). Thus, we suggest that EHMT2 downregulation by SP treatment suppressed cell proliferation via cell apoptosis in CRC cell lines, indicating that EHMT2 is a key protein for SP-induced cell apoptosis.

### TNFAIP1 is a direct target of EHMT2 for the induction of apoptosis of CRC cells

To identify EHMT2 direct target genes, we performed ChIP-seq analysis after treatment with siEHMT2 and focused on the TNFAIP1 gene for SP-induced cell apoptosis because the H3K9 dimethylation status was reduced in the TNFAIP1 promoter regions of the siEHMT2 groups compared with that in the siCont groups (Fig. 4G). To confirm this result, we performed another ChIP assay after treatment with EHMT2 siRNA. We designed two ChIP primers (P1 and P2) targeting the promoter regions of TNFAIP1 and performed ChIP assays using anti-H3K9me2 and anti-H3K9ac antibodies (Fig. 4H). The H3K9 dimethylation status was significantly decreased in the promoter region of the TNFAIP1 gene after EHMT2 knockdown compared with that in the siCont group. In addition, H3K9 acetylation in the promoter region of the TNFAIP1 gene increased, suggesting that EHMT2 directly regulated TNFAIP1 expression by altering chromatin structures (Fig. 4I, J).

### Epigenetic regulation of TNFAIP1 by EHMT2 induces the apoptosis of CRC cells

**Fig. 5:**
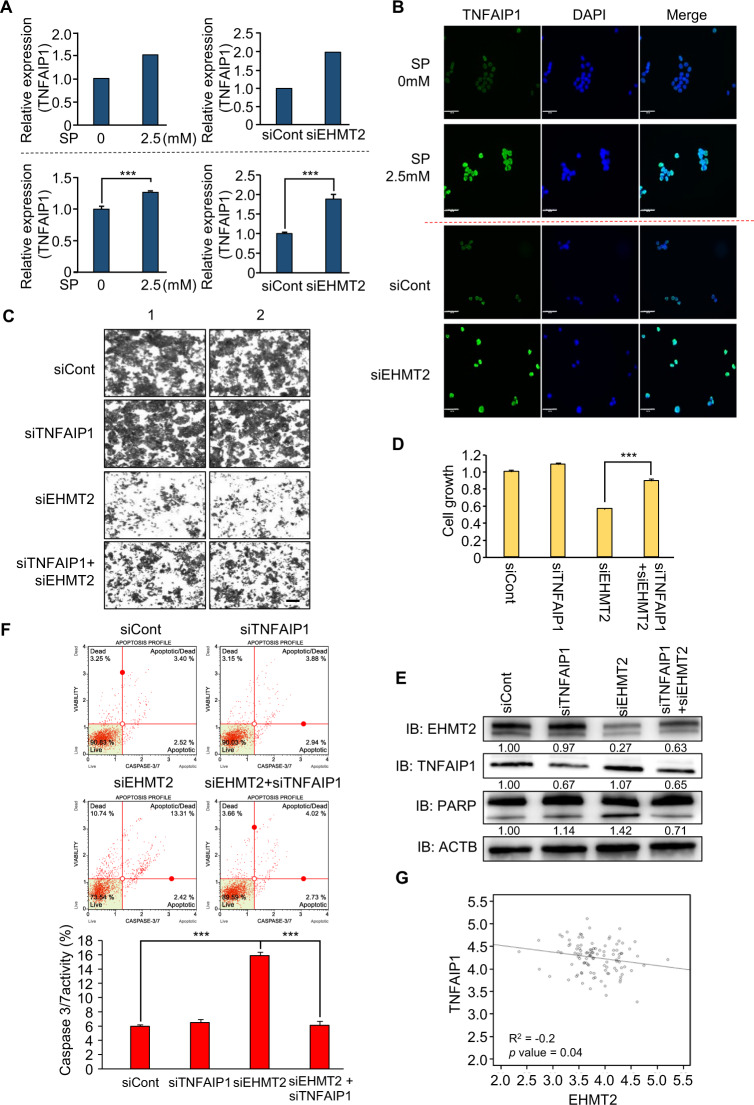
Negative regulation of TNFAIP1 by EHMT2 induces CRC apoptosis. **A** Expression level of TNFAIP1 in RNA-seq results after treatment with SP or siEHMT2 (upper). qRT-PCR analysis of TNFAIP1 after treatment with SP or siEHMT2. Mean ± SD of three independent experiments. *p* values were calculated using Student’s *t*-test (***p < 0.001) (below). **B** Immunocytochemical analysis of TNFAIP1. HCT116 cells treated with SP or siEHMT2 were fixed with 100% methanol and stained with anti-TNFAIP1 (Alexa Fluor 488, green) and DAPI (blue). Scale bar, 50 μm. **C**, **D** Cell growth assay after cotransfection with siTNFAIP1 and siEHMT2 for 48 h. HCT116 cells were fixed in 100% methanol and stained with crystal violet solution. Scale bar, 200 μm (**C**). CCK-8 solution was added to the culture medium, and the cells were incubated for 5 min at 37 °C. The intensity of cell growth was measured using a microplate reader (450 nm). Mean ± SD of three independent experiments. *p* values were calculated using Student’s *t*-test (***p < 0.001) (**D**). **E** Western blot analysis after cotransfection of siEHMT2 and siTNFAIP1 using anti-PARP, anti-EHMT2, and anti-TNFAIP1 antibodies. ACTB was used as the internal control in HCT116 cells. The signal intensities were quantified using ImageJ software. **F** FACS analysis using Muse Caspase 3/7 working solution was performed after cotransfection of siEHMT2 and siTNFAIP1. The upper right panel indicates the apoptotic and dead cell proportions (upper). Quantification of caspase 3/7 activity. Mean ± SD of three independent experiments. *p* values were calculated using Student’s *t*-test (***p < 0.001) (below). **G** Scatter plots of EHMT2 and TNFAIP1 in the GTEx portal. Each dotted line indicates a linear regression line of the expression of EHMT2 and TNFAIP1. *p* values and correlation coefficients (*r*) between two genes were obtained using the Pearson correlation method.

EHMT2 is involved in the negative regulation of gene expression by forming a heterochromatin structure through H3K9 dimethylation [31]. Thus, we selected upregulated genes, which are candidate genes for direct regulation by EHMT2, after EHMT2 knockdown. TNFAIP1 is an early response gene induced by IL-6 and TNFα and is involved in cell apoptosis, DNA repair, and DNA synthesis. In gastric cancer, TNFAIP1 overexpression induced by miR-372 knockdown promoted cell apoptosis by regulating the NF-κB signaling pathway. In HeLa cell lines, overexpressed TNFAIP1 also increased cell apoptosis by downregulating NF-κB expression [32–35]. Thus, we selected TNFAIP1 as the final target of EHMT2-induced apoptosis. The RNA-seq results revealed upregulation of TNFAIP1 after SP treatment and EHMT2 knockdown in CRC cell lines, and the induction of TNFAIP1 was confirmed by qRT-PCR analysis, implying that propionate-induced TNFAIP1 expression by downregulating EHMT2 (Fig. 5A). In ICC analysis, the intensity of TNFAIP1 increased after SP or siEHMT2 treatment compared with that of the control cells (Fig. 5B). Thus, we suggest that the downregulation of EHMT2 induces TNFAIP1 expression at the transcriptional level. Next, to assess the relationship between TNFAIP1 and EHMT2 knockdown, we performed a cell growth assay with CV staining and CCK-8 analysis at the phenotypic level. Growth recovery by cotreatment with EHMT2 and TNFAIP1 siRNAs compared with that of siEHMT2 treatment only was observed, as demonstrated by CV staining and CCK-8 assays (Fig. 5C, D). In western blot analysis, the induction of increased PARP by EHMT2 knockdown was reduced by cotreatment with siEHMT2 and siTNFAIP1 (Fig. 5E). In addition, FACS analysis to estimate caspase 3/7 activation and apoptosis showed that cotreatment with EHMT2 and TNFAIP1 siRNAs decreased caspase 3/7 activity (Fig. 5F). Thus, TNFAIP1 is an effector gene after EHMT2 degradation by SP treatment in CRC cell lines.

Next, to confirm the interaction between EHMT2 and TNFAIP1, we performed correlation analysis using CRC public RNA-seq results derived from the TCGA portal. EHMT2 and TNFAIP1 exhibited a negative correlation (Fig. 5G; *R*^2^ = –0.2; *p* value = 0.04). Thus, we suggest that SP-induced proteasomal degradation of EHMT2 reduces H3K9 dimethylation in the promoter region of TNFAIP1; consequently, apoptosis of CRC cells is increased by TNFAIP1 upregulation.

### Human gut-microbiome-derived bacterial culture medium suppresses the growth of HCT116 cells by upregulating TNFAIP1 expression

**Fig. 6:**
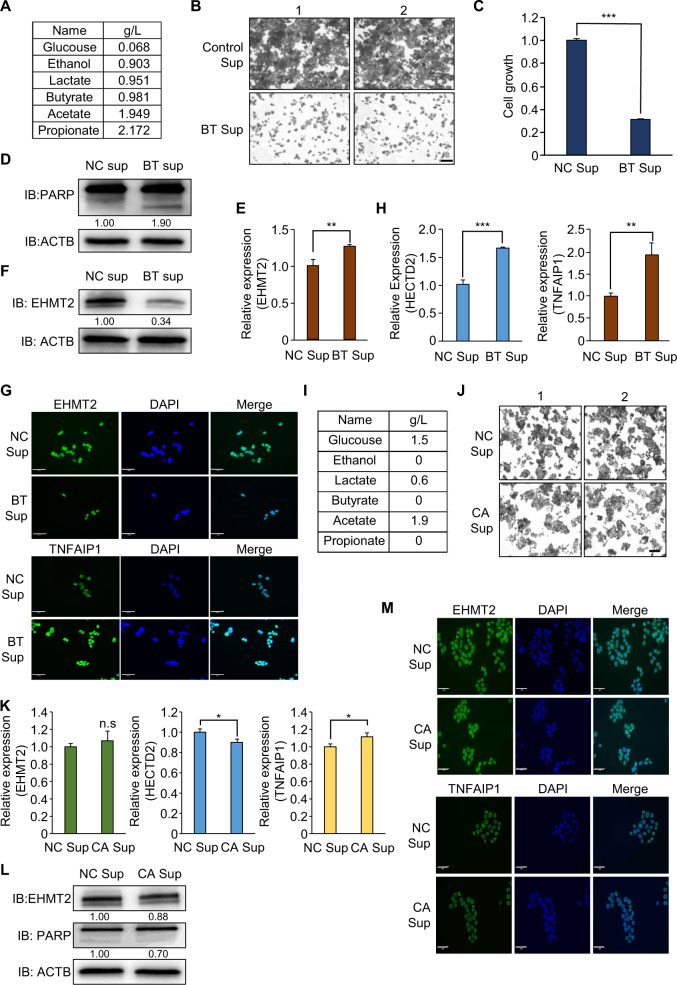
The supernatant of BT culture medium suppresses the growth of HCT116 and LS174T cells via propionate-induced apoptosis. **A** The concentration of metabolites was determined using a high-performance liquid chromatography (HPLC) system (1200 series, Agilent, USA) with a refractive index detector (RID) and an ion-exchange column (300 × 78 mm; Aminex HPX-87H; Bio-Rad, USA). The mobile phase was 2.5 mM H_2_SO_4_, the flow rate was 0.6 ml/min, the column temperature was 65 °C, and the RID was maintained at 45 °C. **B**, **C** Cell growth assay (crystal violet staining and CCK-8 assay) after BT Sup treatment for 48 h. HCT116 cells were fixed in 100% methanol and stained with crystal violet solution. Scale bar, 200 μm. (**B**). CCK-8 solution was added to the culture medium, and the cells were incubated for 5 min at 37 °C. The intensity of cell growth was measured using a microplate reader (450 nm). Mean ± SD of three independent experiments. *p* values were calculated using Student’s *t*-test (***p < 0.001) (**C**). **D** Western blot analysis after BT Sup treatment using anti-PARP. ACTB was used as the internal control in HCT116 cells. The signal intensities were quantified using ImageJ software. **E** qRT-PCR analysis of EHMT2 expression after BT Sup treatment. Mean ± SD of three independent experiments. *p* values were calculated using Student’s *t*-test (**p < 0.01). **F** Western blot analysis after BT sup treatment using anti-EHMT2. ACTB was used as the internal control. The signal intensities were quantified using ImageJ software. **G** Immunocytochemical analysis of EHMT2 and TNFAIP1. HCT116 cells treated with BT Sup were fixed with 100% methanol and stained with anti-EHMT2 and anti-TNFAIP1 (Alexa Fluor 488, green) and DAPI (blue). Scale bar, 50 μm. **H** qRT-PCR analysis of HECTD2 and TNFAIP1 after BT Sup treatment. Mean ± SD of three independent experiments. *p* values were calculated using Student’s *t*-test (***p < 0.001, **p < 0.01). **I** The concentration of metabolites was determined using a high-performance liquid chromatography (HPLC) system. **J** Cell growth assay (crystal violet staining) after CA Sup treatment for 48 h. HCT116 cells were fixed in 100% methanol and stained with crystal violet solution. Scale bar, 200 μm. **K** qRT-PCR analysis of EHMT2, HECTD2, and TNFAIP1 expression after CA Sup treatment. Mean ± SD of three independent experiments. *p* values were calculated using Student’s *t*-test (*p < 0.05, n.s. nonsignificant). **L** Western blot analysis after CA Sup treatment using anti-EHMT2 and anti-PARP antibodies. ACTB was used as the internal control. The signal intensities were quantified using ImageJ software. **M** Immunocytochemical analysis of EHMT2 and TNFAIP1. HCT116 cells treated with CA Sup were fixed with 100% methanol and stained with anti-EHMT2, anti-TNFAIP1 (Alexa Fluor 488, green) and DAPI (blue). Scale bar, 50 μm.

To verify whether human microbiome-derived propionate suppresses CRC growth by downregulating EHMT2, we performed another biochemical analysis using the human microbiome BT. In the colon, the *Bacteroides* genus is a major group of the human microbiome, and BT is the second most commonly isolated bacteria after *B. fragilis* [36–38]. BT is a gram-negative obligate anaerobe. This bacterium is involved in polysaccharide metabolism, such as the hydrolysis of glycosidic bonds in dietary carbohydrates [36]. First, we collected the BT supernatant (Sup) to analyze propionate production via HPLC analysis. In the HPLC results, we confirmed the production of propionate (2.172 g/l) in BT Sup (Fig. 6A). To evaluate the anticancer effect of BT in colon cancer, we added BT Sup to the culture medium of CRC cell lines. Cell growth in the BT Sup treatment group was significantly inhibited compared with that in the control group (media only), as demonstrated by CV staining and CCK assays (Fig. 6B, C). In addition, western blot analysis indicated that cleaved PARP was increased by BT Sup treatment, implying that propionate derived from BT may suppress CRC cell line growth by inducing cell apoptosis (Fig. 6D). Next, to confirm the reduction of EHMT2 expression by BT, we performed western blot analysis and qRT-PCR analysis after BT Sup treatment in HCT116 cells and found that BT Sup treatment reduced EHMT2 expression at the protein level but not at the transcriptional level (Fig. 6E, F). In addition, ICC analysis after BT Sup treatment revealed that the intensity of EHMT2 in BT-treated cells was significantly decreased compared with that in the control cells (Fig. 6G). Furthermore, we observed that BT Sup upregulated HECTD2 and TNFAIP1 expression compared with that in the control group, as shown by propionate treatment in qRT-PCR analysis (Fig. 6H). Finally, in ICC, the expression of TNFAIP1 was significantly increased by BT Sup treatment (Fig. 6G).

Hineebusch et al. demonstrated that treatment with SCFAs (propionate, butyrate) induced histone H4 hyperacetylation by inhibiting HDAC activity [39]. Because butyrate also showed HDAC inhibitor activity similar to propionate, we hypothesized that butyrate in BT Sup might be involved in the HECTD2-EHMT2-TNFAIP1 axis for growth suppression of colon cancer cell lines via the reduction of HECTD2 by inducing H3K27 acetylation, as shown by propionate treatment. To evaluate this hypothesis, we first performed another cell growth assay after treatment with SB in HCT116 cells and found growth suppression by SB treatment in CCK analysis (Supplementary Fig. S6A). In western blot and FACS analyses, we observed the induction of cleaved PARP and caspase 3/7 activity, suggesting that SB treatment also induced cell apoptosis, as shown by SP treatment (Supplementary Fig. S6B, C). To assess the HECTD2-EHMT2-TNFAIP1 axis by SB treatment, we performed qRT-PCR and ICC analysis. SB treatment induced HECTD2 and TNFAIP1 expression (Supplementary Fig. S6D, E). Although EHMT2 expression was slightly increased by SB treatment, western blot and ICC analyses showed that EHMT2 expression was significantly decreased after SB treatment. Next, to assess whether SB treatment affects H3K27 acetylation, we performed western blot analysis to detect H3K27 acetylation after SB treatment in HCT116 cells and found the induction of H3K27 acetylation compared with the control (Supplementary Fig. S6F). In addition, in ChIP analysis with H3K27 acetylation antibody, we clearly found enrichment of the H3K27 acetylation status in the HECTD2 promoter region, as shown with SP treatment (Supplementary Fig. S6G). Finally, we performed a recovery study after cotreatment with SB and siHECTD2 in HCT116 cell lines. The induction of caspase 3/7 activity by SB treatment was decreased by siHECTD2 treatment (Supplementary Fig. S6H). Thus, we suggest that microbiome-induced butyrate inhibits colon cancer growth by regulating the HECTD2-EHMT2-TNFAIP1 axis and HDAC activity, as shown by SP treatment.

As the negative control, we selected CA, which is a gram-positive bacterium in the human intestine [40]. In HPLC analysis, we did not detect propionate and butyrate in the CA supernatant (Fig. 6I). Using CA Sup, we evaluated the HECTD2-EHMT2-TNFAIP1 axis in HCT116 cell lines. In the growth assay using CV staining, no growth inhibition was found after CA sup treatment (Fig. 6J). In addition, in qRT-PCR analysis, although TNFAIP1 expression was slightly increased by CA sup, CA sup treatment did not affect HECTD2 expression (Fig. 6K). Furthermore, western blotting and ICC detected no changes in EHMT2 and TNFAIP1 expression (Fig. 6L, M). Taken together, we suggest that the SCFA-producing microbiome, including BT, in the human body plays an important role in preventing colon cancer.

### EHMT2 is a therapeutic target for CRC

**Fig. 7:**
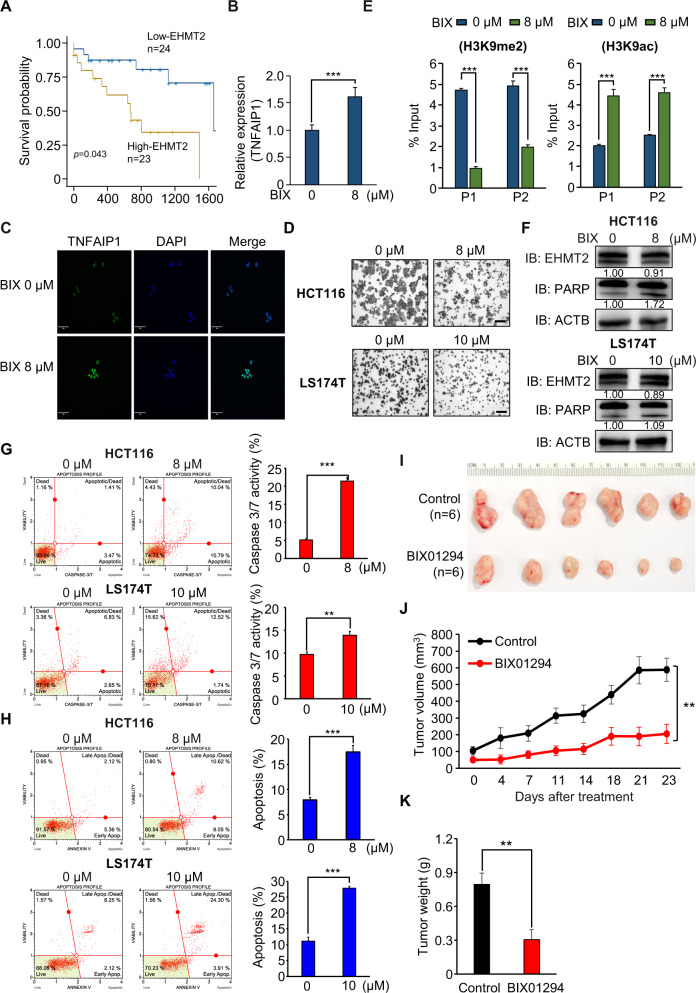
EHMT2 is a therapeutic target for CRC. **A** Kaplan–Meier plot of overall survival in CRC samples derived from the GTEx portal. The survival rate of the low EHMT2 group was significantly increased compared with that of the high EHMT2 group (*p* = 0.043). **B** qRT-PCR analysis of TNFAIP1 after BIX01294 treatment. Mean ± SD of three independent experiments. *p* values were calculated using Student’s *t*-test (***p < 0.001). **C** Immunocytochemical analysis of TNFAIP1. HCT116 cells treated with BIX01294 were fixed with 100% methanol and stained with anti-TNFAIP1 (Alexa Fluor 488, green) and DAPI (blue). Scale bar, 50 μm. **D** Cell growth assay after BIX01294 treatment for 24 h. HCT116 and LS174T cells were fixed in 100% methanol and stained with crystal violet solution. Scale bar, 200 μm. **E** The ChIP assay was performed using anti-H3K9me2 (left) and anti-H3K9ac (right) antibodies. The results are expressed as a percentage of input chromatin compared with the control in HCT116 cells after BIX01294 treatment. Mean ± SD of three independent experiments. *p* values were calculated using Student’s *t*-test (***p < 0.001). **F** Western blot analysis after BIX01294 treatment using anti-PARP and anti-EHMT2 antibodies. ACTB was used as the internal control in HCT116 and LS174T cells. The signal intensities were quantified using ImageJ software. **G** FACS analysis using Muse Caspase 3/7 working solution was performed after BIX01294 treatment. The upper right panel indicates the apoptotic and dead cell proportions (left). Quantification of caspase 3/7 activity. Mean ± SD of three independent experiments. *p* values were calculated using Student’s *t*-tests (***p < 0.001, **p < 0.01) (right). **H** FACS analysis of Annexin V staining was performed after BIX01294 treatment. The lower right and upper right quadrants indicate early apoptosis and late apoptosis, respectively. Quantification of apoptosis. Mean ± SD of three independent experiments. *p* values were calculated using Student’s *t*-test (***p < 0.001) (right). **I**–**K** BIX01294 treatment suppressed xenograft nude mouse tumors. Either control or BIX01294 was intraperitoneally injected three times a week after HCT116 cell implantation. **I** Macroscopic image of tumors on day 23, **J** tumor volumes [*p* values were calculated using two-way ANOVA (***p < 0.001)], and **K** tumor weight [*p* values were calculated using Student’s *t*-test (**p < 0.01)].

Using the RNA-seq results of colon cancer and normal tissues derived from the TCGA portal, we determined the prognostic value of EHMT2 in CRC. The high-expression EHMT2 group showed a poor prognosis compared with the low-expression EHMT2 group (*p* = 0.043; Fig. 7A), indicating that EHMT2 is an attractive therapeutic target for CRC treatment and can be used to prevent CRC. To assess EHMT2 as a therapeutic target of CRC, we used a BIX01294 inhibitor, which is a specific inhibitor of EHMT2 activity [41]. After BIX01294 treatment, TNFAIP1 expression significantly increased by reducing EHMT2 activity, as shown by qRT-PCR and ICC analysis (Fig. 7B, C), and cell growth also decreased, as demonstrated by CV staining (Fig. 7D). In the ChIP assay after treatment with BIX01294, we found a reduction in H3K9me2 status and induction of H3K9ac status in the promoter regions of TNFAIP1 (Fig. 7E). In addition, BIX01294 induced cleaved PARP (western blot analysis) and caspase 3/7 activity (FACS analysis) (Fig. 7F, G). Finally, the population of early and later apoptotic cells increased after BIX01294 treatment in CRC cell lines (Fig. 7H). To verify the CRC growth inhibitory effect of EHMT2 inactivation in vivo, we performed xenograft analysis with BIX01294 and found that the tumor size was reduced in the BIX01294 treatment group compared with that in the control group (Fig. 7I–K).

### Synergistic effect of propionate and BIX01294 in a 3D spheroid model of HCT116 cell lines

**Fig. 8:**
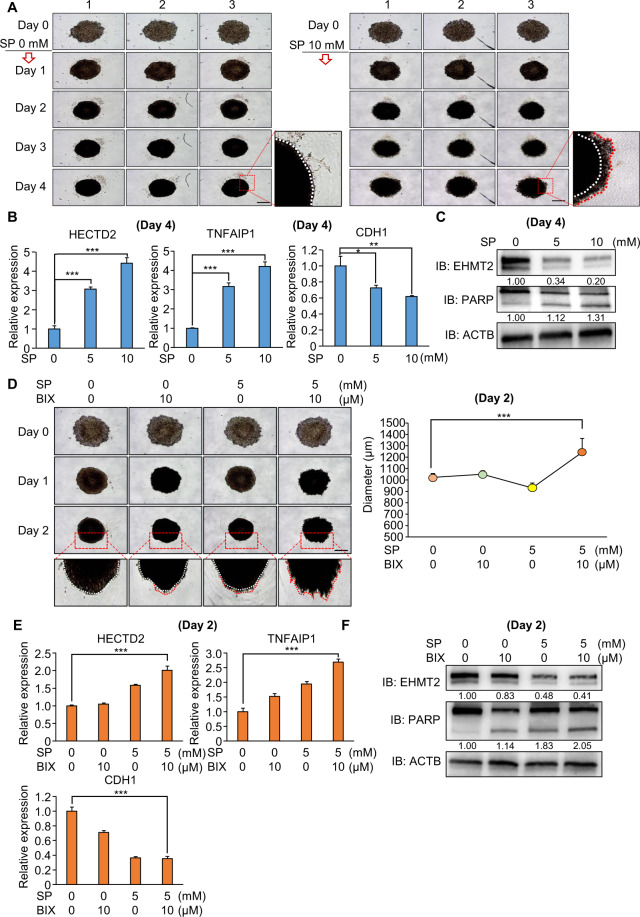
Synergistic effect of SP and BIX01294 in the 3D spheroid model. **A** 3D spheroid formation assay with HCT116 cell lines. Cells treated with SP (0 and 10 mM) were loaded onto ULA plates and incubated for 4 days. The cells were photographed under a microscope each day. Scale bar, 500 μm (left). Enlargement of spheroids at 4 days (right). Red line: dissociated cells. White line: aggregated cells. **B** qRT-PCR analysis of HECTD2, TNFAIP1, and CDH1 at 4 days. Mean ± SD of three independent experiments. *p* values were calculated using Student’s *t*-test (*p < 0.05, **p < 0.01, ***p < 0.001). **C** Western blot analysis after SP treatment using anti-EHMT2 and anti-PARP antibodies. ACTB was used as the internal control. **D** 3D spheroid formation assay with HCT116 cell lines. Cells cotreated with SP and BIX01294 were loaded onto ULA plates and incubated for 2 days. The cells were photographed under a microscope each day. Scale bar, 500 μm. Enlargement of spheroids in 2 days. Red line: dissociated cells. White line: aggregated cells (left). The size of spheroids was measured using ImageJ software (right). Mean ± SD of three independent experiments. *p* values were calculated using Student’s *t*-test (***p < 0.001). **E** qRT-PCR analysis of HECTD2, TNFAIP1, and CDH1 at 2 days. Mean ± SD of three independent experiments. *p* values were calculated using Student’s *t*-test (*p < 0.05, **p < 0.01, ***p < 0.001). **F** Western blot analysis after SP treatment using anti-EHMT2 and anti-PARP antibodies. ACTB was used as the internal control.

To validate the propionate effect and synergistic effect of cotreatment with SP and an EHMT2 inhibitor (BIX01294) in colon cancer, we used a 3D spheroid model system using a ULA plate. The 3D spheroid model system reflects the tumor physiological conditions and complexity of the tumor structure, implying that the 3D spheroid model is a useful model for target study and anticancer drug development for cancer treatment. First, in ULA plate culture in HCT116 cells, we detected the spheroids of HCT116 cells on day 4. However, the aggregation of spheroids was loosened by SP treatment compared with that of the negative control (PBS only; Fig. 8A). To assess the dissociation of spheroids, we used E-cadherin (CDH1), which is a marker of cell aggregation, and the expression of E-cadherin was decreased by dissociation of the spheroids [42]. In qRT-PCR analysis, the expression of E-cadherin was decreased in the SP treatment group compared with the control group. In addition, depending on SP treatment, we observed HECTD2 and TNFAIP1 upregulation, as shown in the 2D culture system (Fig. 8B). Next, to verify the downregulation of EHMT2 by SP, we performed western blot analysis and found a reduction in EHMT2 expression after SP treatment. In addition, cleaved PARP was increased by SP treatment compared with PBS, as shown in the 2D culture system (Fig. 8C). Thus, we confirmed the anticancer effect of propionate in the 3D culture system, suggesting that the propionate-generating microbiome may control the prevention of colon cancer development.

Next, to evaluate the synergistic effect of cotreatment with SP and an EHMT2 inhibitor, we used a 3D culture system with HCT116 cell lines. In bright field images, we found the strongest dissociation of the spheroids in the SP/BIX01294 cotreatment group compared with that in the control and single-treatment groups (SP or BIX01294) on day 2, suggesting a synergistic effect between day 4 (SP only) and day 2 (SP and BIX01294) (Fig. 8D). qRT-PCR showed that the expression of HECTD2 and TNFAIP1 significantly increased in the cotreatment group (Fig. 8E). In addition, in the cleaved PARP status, the strongest induction was found in the cotreatment group compared with that in the other groups (Fig. 8F). Taken together, we suggest that, for colon cancer treatment, if patients use an EHMT2 inhibitor while ingesting a propionate-generating microbiome, we expect a higher anticancer effect.

## Discussion

SCFAs such as butyrate and propionate act as HDAC inhibitors to affect the transcriptional regulation of gene expression [43, 44]. After treatment with SCFAs in CRC, we observed upregulation of apoptosis-related genes via the induction of histone acetylation on promoter regions, implying that SCFAs are useful anticancer inhibitors. In addition, the microbiome that produces SCFAs has also been investigated for its effects on health [10]. To date, gene regulation by SCFAs has mainly been mediated at the transcriptional level, but no reports have examined the relationship between posttranslational modification and SCFA treatment in colon cancer. Therefore, in this study, we first suggested a novel function of propionate in CRC cell lines, according to the enhancement of EHMT2 proteasomal degradation by propionate in these cells (Fig. 2).

For rapid elimination of proteins in cells, the ubiquitin-proteasome pathway is the most effective process for small-peptide recycling during new protein synthesis. In the proteasomal degradation pathway, polyubiquitinated proteins bound to the proteasome by E3 complex proteins are unfolded and degraded. Recently, several papers have reported that the proteasome itself is a potential therapeutic target in cancer treatment [45]. After cotreatment with SP and MG132 in CRC cell lines, EHMT2 expression was rescued from the reduction induced by SP only. In addition, cotreatment with CHX and SP showed more rapid degradation of EHMT2 expression than SP-only treatment, suggesting that SP treatment induced EHMT2 proteasomal degradation (Fig. 2F, G). Using RNA-seq and qRT-PCR analysis, we observed HECTD2 upregulation by SP and BT Sup treatment to promote EHMT2 degradation. Using GENT analysis (http://gent2.appex.kr/gent2/), we found that the expression level of HECTD2 was not different between the control and CRC samples (Supplementary Fig. S7), implying that HECTD2 upregulation by propionate in CRC promotes EHMT2 proteasomal degradation by inducing polyubiquitination.

To assess the anticancer activity of SP in CRC cell lines, we selected the appropriate SP concentration (2.5~5 mM) to perform biochemical analysis and observed SP-induced apoptosis in HCT116 and LS174T cell lines. SCFAs such as propionate, butyrate, and acetate are microbial fermentation products generated using dietary fibers in the human colon. In the lumen of the human colon, the concentrations of propionate and acetate are 10~100 mM [46]; thus, we considered that the concentration of propionate used in this study was appropriate to test the anticancer effect of this molecule. In addition, using hPSC-derived intestinal organoids, we performed a cell growth assay. In Fig. 1I, J, 2.5 and 5 mM SP did not affect the viability of hIO cells; in other words, HCT116 cells were significantly suppressed in the propionate group.

In this study, we used BT Sup to evaluate the SP effects in CRC cell lines. The HPLC results of BT Sup revealed various metabolites. We evaluated the SP effect in BT sup by calculating the IC_50_ values of lactate, acetate, and butyrate in HCT116 cell lines; they were 29.21, 27.51, and 1.38 mM, respectively. In western blot analysis, only butyrate treatment induced cleaved PARP in HCT116 cell lines (Supplementary Fig. S8). Thus, we suggest that propionate and butyrate of BT Sup inhibited the proliferation of HCT116 cell lines by regulating the HECTD2-EHMT2-TNFAIP1 axis. BT only could not represent the gut microbiota. However, because the MOA of SCFAs in colon cancer is not well understood, in this study, we focused on the exact molecular pathway for propionate-induced apoptosis in colon cancer. Finally, we defined the epigenetic regulation and molecular target for propionate-induced apoptosis in colon cancer. Because we must further evaluate our mechanism using propionate-producing bacteria, we selected the BT supernatant and confirmed the HECTD2-EHMT2-TNFAIP1 axis by BT sup treatment, implying that propionate-producing bacteria in the gut microbiota, including BT, may suppress the proliferation of colon cancer and induce cell apoptosis.

To assess the synergistic effect of SP and BIX01294, we used a 3D spheroid model system and found that SP-induced regulation of the HECTD2-EHMT2-TNFAIP1 axis increased CRC apoptosis. In the SP single treatment, we observed cell apoptosis at 4 days. However, in cotreatment with SP and BIX01294, cleaved PARP more increased at 2 days. Thus, we suggest that downregulation of EHMT2 by SP and inhibition of EHMT2 activity by BIX01294 might have a synergistic effect on the suppression of the CRC 3D spheroid model. However, although 3D models can mimic the tumor physiological conditions and complexity of the tumor structure, there are limitations to a 3D spheroid model system to confirm the in vitro results. Thus, to overcome the limitations of propionate-induced CRC apoptosis, further in vivo studies are needed to validate the direct connection between microbially synthesized propionate in the gut and colon cancer in the gut.

To date, the clinical application of propionate in cancer has not been performed, although propionate has shown anticancer effects in several types of cancers, and SCFAs play a critical role in the maintenance of epithelial cells for colonic health [46]. Therefore, although a clinical study with propionate has not been performed, based on the in vitro results for propionate-induced CRC suppression, we suggest the following: (1) the intake of the useful microbiome to produce propionate to inhibit CRC growth; (2) the use of dietary fiber for propionate production; and (3) the screening of a novel microbiome that highly produces propionate.

**Fig. 9:**
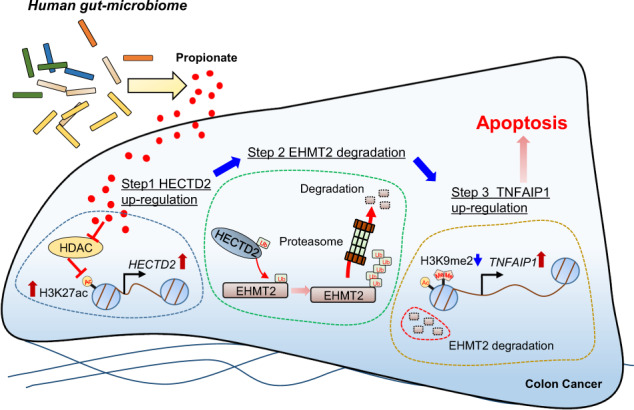
Schematic summary of SP-induced CRC apoptosis. Human gut-microbiome-derived propionate decreased the protein stability of EHMT2 by up-regulation of HECTD2. Subsequently, TNFAIP1 as a novel direct target of EHMT2 induced the apoptosis of colon cancer.

In summary, propionate in colon cancer decreased the protein stability of EHMT2 by promoting proteasomal degradation. Thus, a reduction in EHMT2 expression also decreased the level of histone H3K9 dimethylation in the promoter region of TNFAIP1. Finally, TNFAIP1 upregulation by epigenetic regulation suppressed CRC proliferation via the cell apoptosis machinery (Fig. 9). Thus, the results of the present study suggest the importance of the human microbiome in the colon to prevent colon cancer, and SCFAs such as propionate and HMT-specific inhibitors may show synergistic effects for colon cancer treatment. In addition, dietary therapy for CRC patients is also emphasized to help cure CRC.

## Supplementary information

Supplementary Figures

Supplementary Tables
