# Supplementary material for: Human gut-microbiome-derived propionate coordinates proteasomal degradation via HECTD2 upregulation to target EHMT2 in colorectal cancer
Source: ISME J. 2022 Jan 1;16(5):1205–21. doi: 10.1038/s41396-021-01119-1 (PMC9038766; doi:10.1038/s41396-021-01119-1)
Supplement: Supplementary file 1 — Supplementary Figures [file 41396_2021_1119_MOESM1_ESM.docx]

**
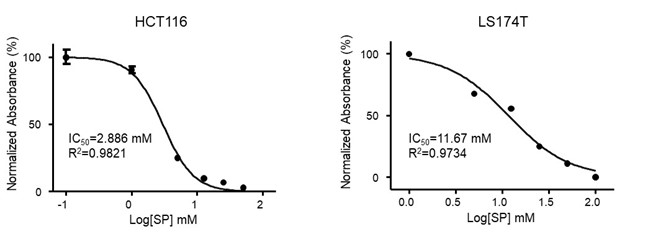
**

**Supplementary Fig. S1** IC_50_ concentration analysis after treatment of SP. HCT116 and LS174T cells were seeded at a density of 5X 10^3^cells/well and 1X10^4^cells/well,in 96-well microtitre plates, respectively. After 24 h incubation. SP was treated and cultured for an 48 h. Cell viability was assessed using CCK-8 assay. CCK8 solution were added to each well and incubated with 5% CO2 at 37˚C for 15min. Each value was normalized to cells treated with PBS and the IC50 values are calculated using Graphpad Prism software.

**
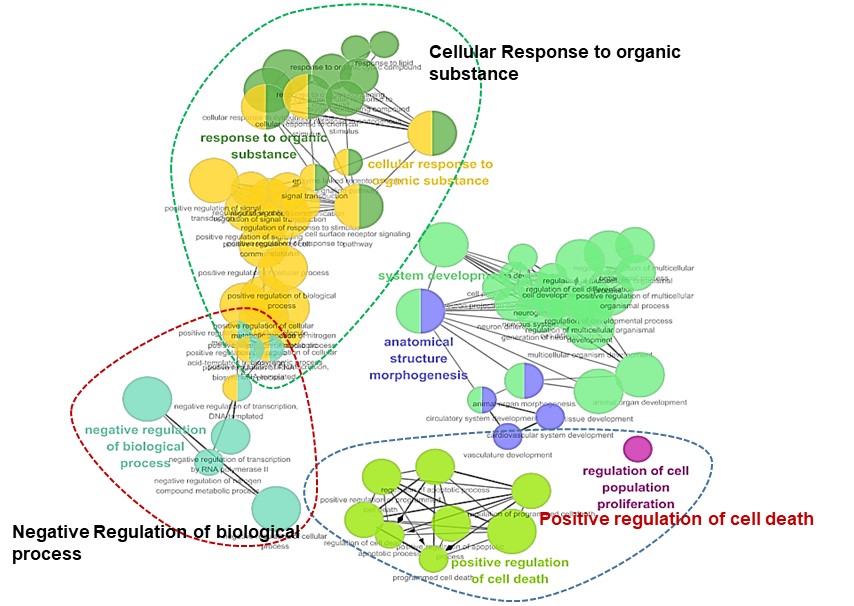
**

**Supplementary Fig. S2** GO pathway term enrichment networks. GO pathway term networks with 872 genes functionally grouped by ClueGO.

**
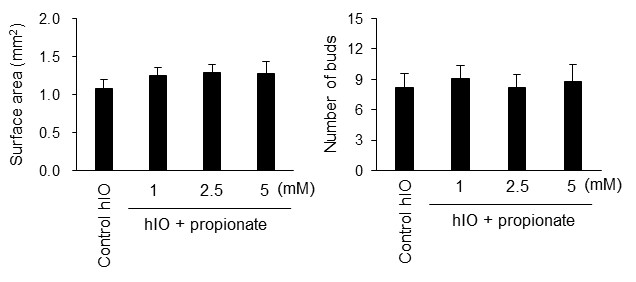
**

**Supplementary Fig. S3** Quantitative assessment of the surface area (n=10, left bottom) (left) and number of budding structure (n=10, right bottom) of hIOs (right) after treatment of propionate.

**
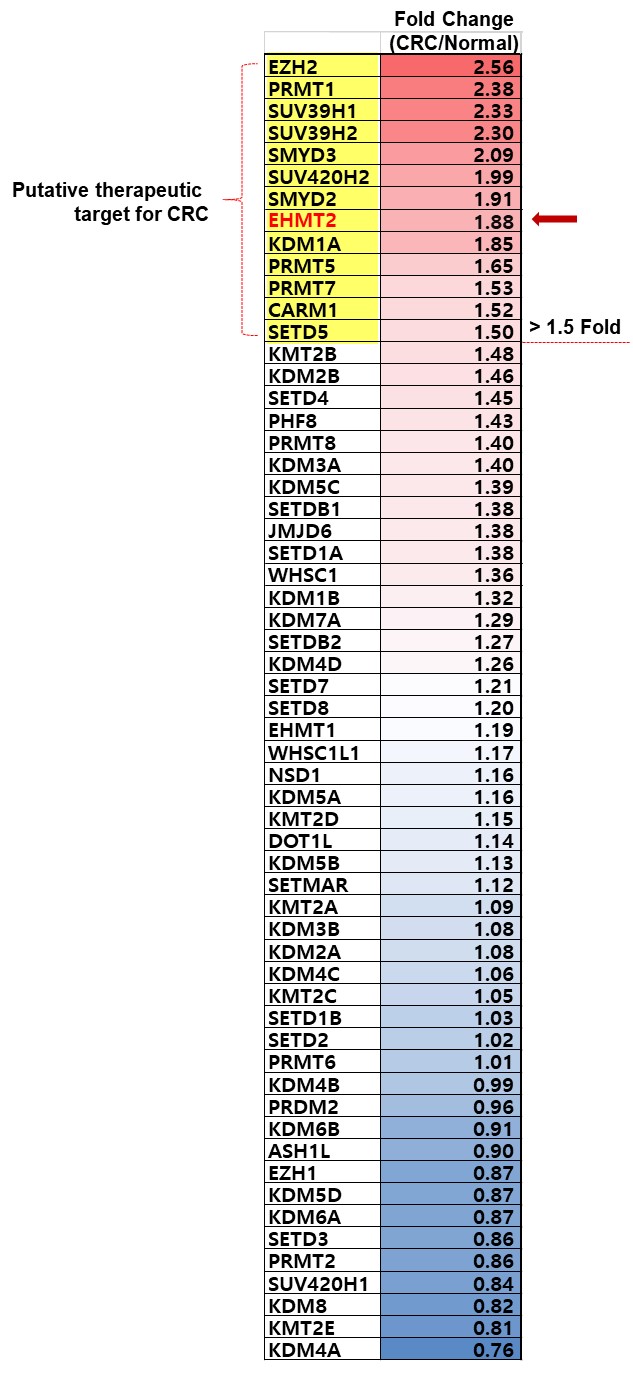
**

**Supplementary Fig. S4** Heat map of histone methyltransferase and demethylase between normal and colon cancer samples derived from TCGA

**
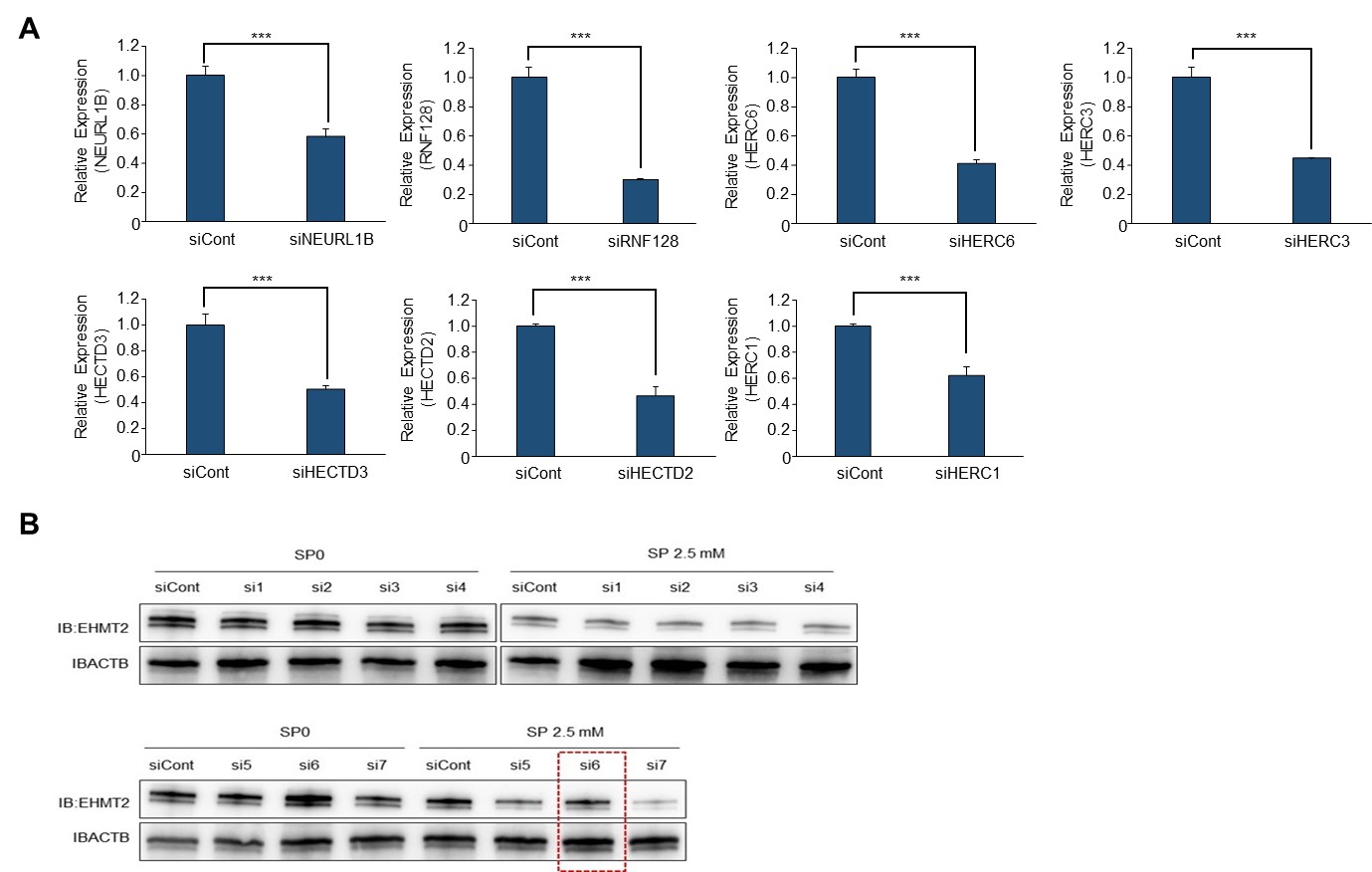
**

**Supplementary Fig. S5 Screening for identification of E3 ligase for EHMT2 degradation**

**a,** qRT-PCR analysis of candidate genes expression after treatment with E3 ligase specific siRNAs and siControl. Mean ±SD of three independent experiments. *p* values were calculated using Student’s *t*-tests (****p* < 0.001). **b,** Western blot analysis of EHMT2 after treatment of siNEURL1B, siRNF128, siHERC6, siHERC3, siHECTD3, siHECTD2, and HERC1 and SP for 48h. ACTB was used as the internal control. (siCont: siRNA Control, si1:NEURL1B, si2:RNF128, si3:HERC6, si4:HERC3, si5:HECTD3, si6:HECTD2, si7:HERC1)

**
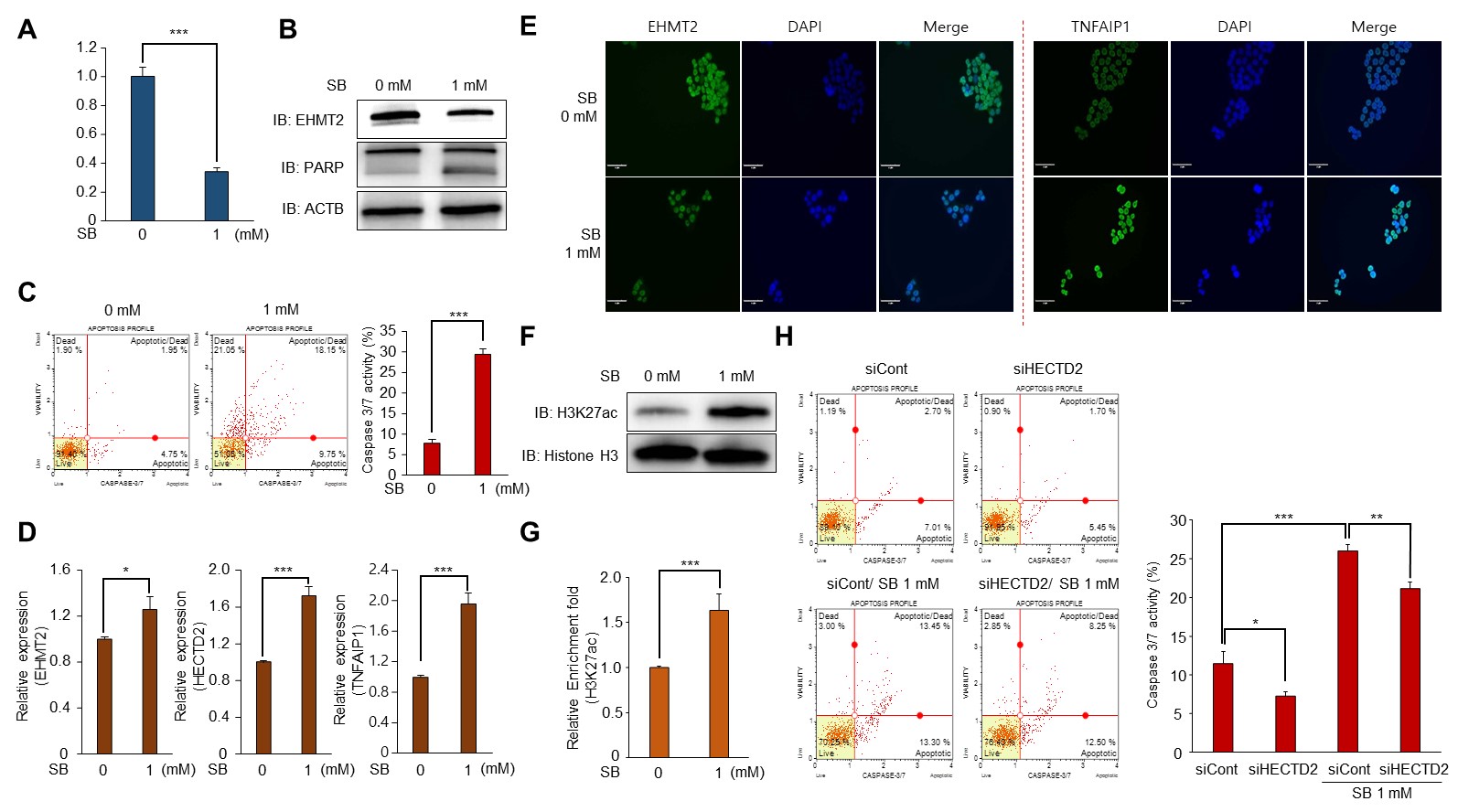
**

**Supplementary Fig. S6 Sodium butyrate (SB) treatment induces EHMT2 degradation via up-regulation of HECTD2. a** Cell growth assay after SB treatment for 24 h. HCT116 cells were incubated for 5 min at 37°C after adding CCK-8 solution. The intensity of cell growth was measured by a microplate reader (450 nm). Mean ±SD of three independent experiments. *p* values were calculated using Student’s *t*-tests (****p* < 0.001). **b** Western blot analysis after SB treatment using anti-PARP and -EHMT2 antiboies . ACTB was used as the internal control in HCT116. **c** FACS analysis using Muse Caspase-3/7 working solution was performed after treatment with SB. The upper right panel indicates the apoptotic and dead cell proportions (left). Quantification of caspase-3/7 activity. Mean ±SD of three independent experiments. *p* values were calculated using Student’s *t*-tests (****p* < 0.001, ***p* < 0.01) (right). **d** qRT-PCR analysis of *EHMT2, HECTD2,* and *TNFAIP1* expression after treatment with SB for 24 h. Mean ±SD of three independent experiments. *p* values were calculated using Student’s *t*-tests (**p* < 0.05, ****p* < 0.001). **e** Immunocytochemical analysis of EHMT2 and TNFAIP1. HCT116 cells treated with SB were fixed with 100% methanol and stained with anti-EHMT2 and -TNFAIP1 (Alexa Fluor 488, green) and DAPI (blue). Scale bar, 50 μm. ). **f** Western blot analysis of histone H3K27 acetylation after treatment with SB for 24 h in HCT116 cells. Histone H3 was used as the internal control. **g** The ChIP assay was performed using anti-H3K27ac antibody. The result is shown as an enrichment fold of input chromatin compared to the control in HCT116 cells after treatment with SB. Mean ±SD of three independent experiments. *p* values were calculated using Student’s *t*-tests. (***, *P* < 0.001). **h** FACS analysis using Muse Caspase-3/7 working solution was performed after cotreatment with HECTD2 knockdown and SB in HCT116 cells. The upper right panel indicates the apoptotic and dead cell proportions (upper) (left). Quantification of caspase-3/7 activity. Mean ±SD of three independent experiments. *p* values were calculated using Student’s *t*-tests (**p* < 0.05, ***p* < 0.01, ****p* < 0.001) (right).

**
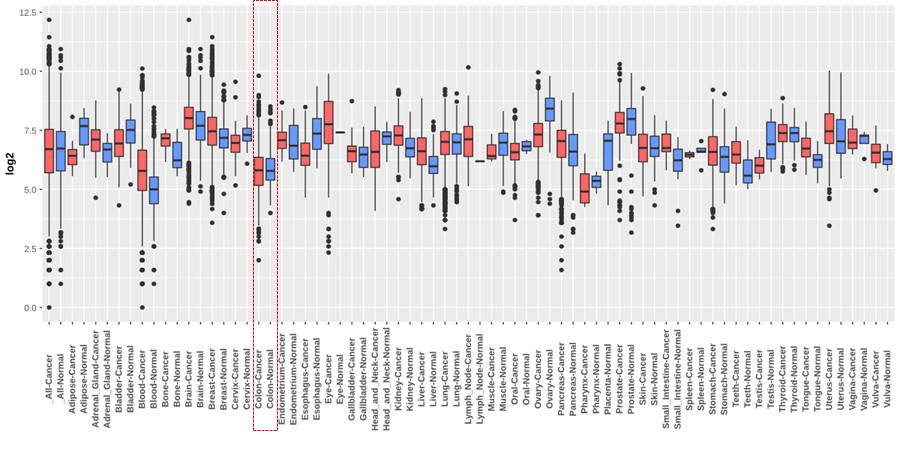
**

**Supplementary Fig. S7 Expression of HECTD2 in several types of cancer.**

Tissue-wide expression profile of *HECTD2* in several types of cancer and normal tissues using GENT2 analysis (http://gene2.appex.kr/gent2/).

**
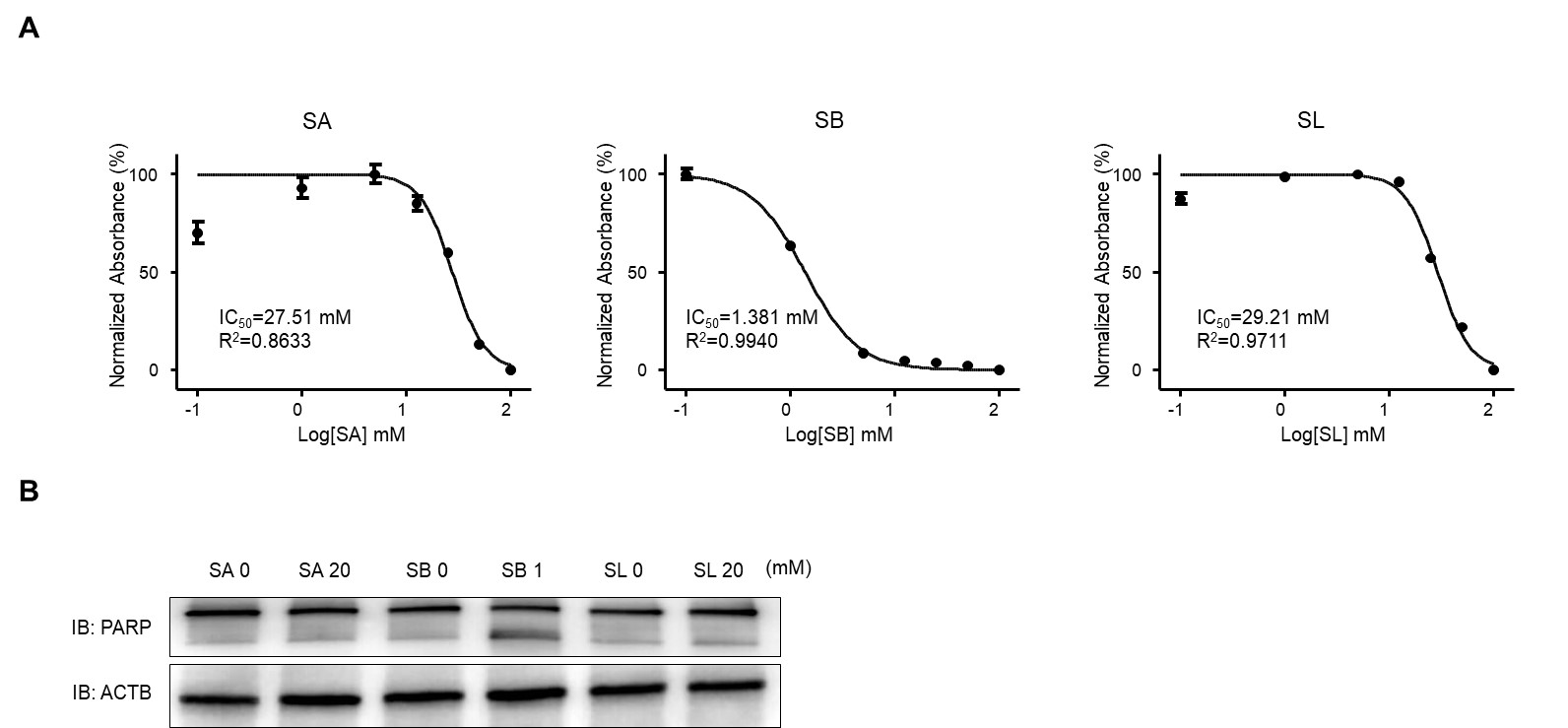
**

**Supplementary Fig. S8 Growth analysis with sodium acetate (SA), sodium lactate (SL), and sodium butyrate (SB) in HCT116 cell lines.**

**a** IC_50_ concentration analysis after treatment of SA, SB SL. HCT116 cells were seeded at a density of 5X10^3^cells/well in 96-well microtitre plates. After 24 h incubation. SA, SB, SL were treated and cultured for an 24 h. Cell viability was assessed using CCK-8 assay. CCK8 solution were added to each well and incubated with 5% CO2 at 37˚C for 15min. Each value was normalized to cells treated with PBS and the IC50 values are calculated using Graphpad Prism software. **b** Western blot analysis after SA, SB, and SL treatment using anti-PARP antibody . ACTB was used as the internal control in HCT116.
