# Supplementary material for: Human gut-microbiome-derived propionate coordinates proteasomal degradation via HECTD2 upregulation to target EHMT2 in colorectal cancer
Source: ISME J. 2022 Jan 1;16(5):1205–21. doi: 10.1038/s41396-021-01119-1 (PMC9038766; doi:10.1038/s41396-021-01119-1)
Supplement: Supplementary file 2 — Supplementary Tables [file 41396_2021_1119_MOESM2_ESM.docx]

**Supplementary Table. S1** siRNA target sequences used in gene knockdown.

| **siRNA name** | **siRNA sequence** |
| --- | --- |
| siCont | AUGAACGUGAAUUGCUCAATT |
| siEHMT2 | GCAAAUAUUUCACCUGCCATT |
| siTNFAIP1 | CUCUCAGGUCAGGUACCUUTT |
| siHECTD2 | CAUUGAAGACUCUGGGAUUTT |
| siNEURL1B | GAGAGAGAAUGAACGUGUTT |
| siRNF128 | CUAAUGACAGAGUAAGUAATT |
| siHERC6 | CAGAUAGAUUGUGGAAGUUTT |
| siHERC3 | CUCCAUGAAUGAUGAGGUUTT |
| siHECTD3 | CUGUACAAAGGCUACUCUATT |
| siHERC1 | CUGUGAUAGAUGAGCUUCATT |

**Supplementary Table. S2** Oligonucleotide primers used in semiquantitative reverse transcription PCR and quantitative real-time PCR.

| **Gene** | **Forward Primer** | **Reverse Primer** |
| --- | --- | --- |
| EHMT2 | 5'-GAGAACATCTGCCTGCACTG-3' | 5'-GTTGACAGCATGGAGGTCAC-3' |
| TNFAIP1 | 5'-CATCACATCCCTAAAGGAGGAGG-3' | 5'-AGCAGGTGGTCGTCAGAGTT-3' |
| HECTD2 | 5'-AGTTCACCTGCACATCTTGTTT-3' | 5'-GCCTTCATTTCGGATGATGATGC-3' |
| CDH1 | 5’-CGAGAGCTACACGTTCACGG-3’ | 5’-GGGTGTCGAGGGAAAAATAGG-3’ |
| ACTB | 5'-ACTCTTCCAGCCTTCCTTCC-3' | 5'-CAATGCCAGGGTACATGGTG-3' |
| NEURL1B | 5'-ACAGCAGCTTCCAAGACACA-3' | 5'-GTTGGGCAGGCTGTAGTAGG-3' |
| RNF128 | 5'-CATGGCCCTTGGGTGAATCA-3' | 5'-CACAGTTGCCGCCGTAATAAT-3' |
| HERC6 | 5'-CCACTCCCTGGCATTATCAAAA-3' | 5'-GCCAAACGAAGTCCCACAGA-3' |
| HERC3 | 5'-TGTTGGGGATATTGGTCTCTGG-3' | 5'-CCCTTGGTGTTCAAACCACAT-3' |
| HECTD3 | 5'-CATCGCCTGGGATCGAGAC-3' | 5'-CGCACTCGTAGGTCCATGTC-3' |
| HERC1 | 5'-TTTGCCCCAACAAGTTTTATGC-3' | 5'-GGAGTACAAGCAGTCGTTTTCT-3' |

**Supplementary Table. S3** Oligonucleotide primers used in ChIP assay.

| **Name** | **Forward** | **Reverse** |
| --- | --- | --- |
| HECTD2 | 5’-CAGCCCCAGCAACACTGAG-3’ | 5’-CTTCTCGCGCTCTGACTCCTTC-3’ |
| TNFAIP1-P1 | 5’-CTGGCAGCCGAACACAAGT-3’ | 5’-CCAAGCCAGATTCATGGGAGT-3’ |
| TNFAIP1-P2 | 5’-ACTCCCATGAATCTGGCTTGG-3’ | 5’-GCTCAGATGCTCAGACACGC-3’ |
